# Artificial Intelligence for Prognostic Scores in Oncology: a Benchmarking Study

**DOI:** 10.3389/frai.2021.625573

**Published:** 2021-04-16

**Authors:** Hugo Loureiro, Tim Becker, Anna Bauer-Mehren, Narges Ahmidi, Janick Weberpals

**Affiliations:** ^1^ Data Science, Pharmaceutical Research and Early Development Informatics (pREDi), Roche Innovation Center Munich (RICM), Penzberg, Germany; ^2^ Institute of Computational Biology, Helmholtz Zentrum Munich, Munich, Germany; ^3^ TUM School of Life Sciences Weihenstephan, Technical University of Munich, Freising, Germany

**Keywords:** electronic health records, machine learning, prognostic scores, real world data, survival analyisis

## Abstract

**Introduction:** Prognostic scores are important tools in oncology to facilitate clinical decision-making based on patient characteristics. To date, classic survival analysis using Cox proportional hazards regression has been employed in the development of these prognostic scores. With the advance of analytical models, this study aimed to determine if more complex machine-learning algorithms could outperform classical survival analysis methods.

**Methods:** In this benchmarking study, two datasets were used to develop and compare different prognostic models for overall survival in pan-cancer populations: a nationwide EHR-derived de-identified database for training and in-sample testing and the OAK (phase III clinical trial) dataset for out-of-sample testing. A real-world database comprised 136K first-line treated cancer patients across multiple cancer types and was split into a 90% training and 10% testing dataset, respectively. The OAK dataset comprised 1,187 patients diagnosed with non-small cell lung cancer. To assess the effect of the covariate number on prognostic performance, we formed three feature sets with 27, 44 and 88 covariates. In terms of methods, we benchmarked ROPRO, a prognostic score based on the Cox model, against eight complex machine-learning models: regularized Cox, Random Survival Forests (RSF), Gradient Boosting (GB), DeepSurv (DS), Autoencoder (AE) and Super Learner (SL). The C-index was used as the performance metric to compare different models.

**Results:** For in-sample testing on the real-world database the resulting C-index [95% CI] values for RSF 0.720 [0.716, 0.725], GB 0.722 [0.718, 0.727], DS 0.721 [0.717, 0.726] and lastly, SL 0.723 [0.718, 0.728] showed significantly better performance as compared to ROPRO 0.701 [0.696, 0.706]. Similar results were derived across all feature sets. However, for the out-of-sample validation on OAK, the stronger performance of the more complex models was not apparent anymore. Consistently, the increase in the number of prognostic covariates did not lead to an increase in model performance.

**Discussion:** The stronger performance of the more complex models did not generalize when applied to an out-of-sample dataset. We hypothesize that future research may benefit by adding multimodal data to exploit advantages of more complex models.

## Introduction

With an estimated incidence of 18.1 million new cases and 9.6 million deaths worldwide in 2018, cancer is still one of the biggest healthcare challenges today (Ferlay et al., 2019). New paradigms such as cancer immunotherapy have led to an increase in survival for several hematological (Sant et al., 2014) and solid tumors (Pulte et al., 2019). Still, drug development in general, including in oncology, suffers from a high attrition rate. Most drugs (97%) fail during early development phases, a process that is both time-consuming (median duration of phase one clinical is 1.6 years) and costly (as much as $42,000 per patient) (Fogel, 2018; Wong et al., 2019). One of the reasons for such failures may be rooted in a suboptimal enrollment of patients in clinical trials. As a consequence, patients may dropout early due to adverse events, lack of tolerability and/or lack of efficacy which might lead to an early failure of potentially effective drugs (Fogel, 2018). In this context, an accurate characterization of the patients’ recovery (or response to medications) given their prognostic factors is key. Currently, the patients’ prognostic factors are used to determine 1) clinical trial eligibility, 2) toxicity monitoring and 3) treatment decisions. Furthermore, prognostic factors allow us to gain a deeper understanding of disease biology and thus may contribute to the development of more effective treatments (Bhimani et al., 2019).

To date, several prognostic scores in oncology have been published, such as the Royal Marsden Hospital Score (Arkenau et al., 2009), the international prognostic index (International Non-Hodgkin’s Lymphoma Prognostic Factors Project, 1993), the IMDC risk model (Ko et al., 2015) or the Glasgow prognostic score (Kinoshita et al., 2013). Due to prior lack of access to large-scale patient data, the previous prognostic scores were significantly limited on the modeling approaches. Additionally, previous databases also usually contained a small number of covariates, which typically were cast into a simple counting scheme (number of covariates above a threshold).

As a major enhancement, the ROPRO was introduced recently (Becker et al., 2020). The ROPRO is a new pan-cancer prognostic score developed from more than 125k patients in the EHR-derived de-identified database which consists of 27 highly prognostic covariates for overall survival. This prognostic score is based on the Cox proportional hazards model (in the following referred to as Cox model) (Becker et al., 2020), a widely used survival analysis model. In (Becker et al., 2020), ROPRO showed an increased prognostic power when compared to the aforementioned scores and was validated in independent clinical data. In general, the Cox model cannot model nonlinearities or interaction effects, unless all of these effects are explicitly specified (Harrell et al., 1996). While the ROPRO is a multivariate model it does not include covariate interactions and possibly could have missed nonlinearities in the covariates.

To overcome the Cox model’s limitations, recent models such as the regularized Cox model (Tibshirani 1997; Simon et al., 2011), random survival forests (Ishwaran et al., 2008), gradient boosting (Ridgeway 1999) and DeepSurv (Katzman et al., 2018) a deep neural network-modified version of the Cox model have been introduced.

Several studies (Chen et al., 2019; Christodoulou et al., 2019; Desai et al., 2020; Kim et al., 2019; Steele et al., 2018) have been published that compare the prognostic/predictive performance of some of these new survival models. Still, there remains the need for a more systematic and direct comparison. Hence, the objective of this study is to compare the prediction performance of a set of models with respect to model complexity and automated covariate selection. We aimed to address model complexity by implementing more complex survival models (regularized Cox (Tibshirani 1997; Simon et al., 2011), Random Survival Forests (Ishwaran et al., 2008), Gradient Boosting (Ridgeway 1999), DeepSurv (Katzman et al., 2018), a new autoencoder based model (Goodfellow et al., 2016) and Super Learner (van der Laan et al., 2007)) and compared them against the classical model (ROPRO (Becker et al., 2020)). To address the automated covariate selection, we investigated whether an increase in the covariate number, even though not present for all patients, led to an increase in model performance.

## Materials and Methods

### Datasets

In this study we used two databases: 1) the nationwide Flatiron Health (FH) electronic health record (EHR)-derived de-identified database and 2) OAK clinical trial database. During the study period, the FH database included de-identified patient-level structured and unstructured data, curated via technology-enabled abstraction (Birnbaum et al., 2020; Ma et al., 2020) and includes data from over 280 cancer clinics (∼800 sites of care); Institutional Review Board approval of the FH study protocol was obtained prior to study conduct, and included a waiver of informed consent. The OAK dataset was derived from a phase III clinical trial (Rittmeyer et al., 2017) that evaluated the efficacy and safety of Atezolizumab monotherapy against a Docetaxel monotherapy in 1,187 patients with locally advanced or metastatic non-small cell lung cancer (NSCLC) after the failure of platinum based chemotherapy.

From FH we derived a cohort with 136,719 patients across 18 different primary cancers (Table 1). The majority of patients were diagnosed with advanced non-small cell lung cancer (38,201–26.7%), followed by metastatic colorectal cancer (16,788–12.1%) and metastatic breast cancer (14,429–10.4%). We randomly split the samples in the FH dataset into train (90% - 121,644) and in-sample test (10% - 15,075) sets. In case the model required a validation dataset (e.g., neural network based models), the training set was further divided into subsets of 90% for training and 10% for validation. The OAK study (1,187 patients) was used exclusively for out-of-sample testing.

**TABLE 1:** Number of patients per cohort in the FH dataset. Includes both train and test datasets.

Cohort	Patient number
Advanced endometrial	1,641
Advanced melanoma	4,332
Advanced non-small-cell lung cancer	38,201
Acute myeloid leukemia	2,232
Bladder cancer	5,363
Chronic lymphocytic leukemia	9,544
Diffuse large B-cell lymphoma	3,969
Breast cancer	655
Follicular cancer	1,958
Gastric cancer	6,212
Head and neck cancer	4,917
Metastatic breast cancer	14,429
Metastatic colorectal cancer	16,788
Metastatic renal cell carcinoma	5,116
Multiple myeloma	7,293
Ovarian cancer	4,407
Pancreatic cancer	6,212
Small-cell lung cancer	4,918

In terms of covariates used per sample, we created three feature sets with differing numbers of covariates that could be used for modeling by the respective method. The first feature set contained 27 covariates of FH inspired from (Becker et al., 2020) (Table 2). The second feature set consisted of 44 covariates that were present in at least 30% of patients in FH, and the third feature set comprised almost all covariates (88 covariates present for at least 1% of the FH patients). The 88 and 44 feature sets included all the covariates of the 44 and 27 feature sets, respectively (a complete list of the covariates in each set is available in Supplementary Table S1). The OAK dataset contained all the covariates used in the 27 covariates feature set except oxygen saturation in blood. In the 44 and 88 feature sets it was in addition lacking information on some covariates as compared to the FH dataset (for a complete list see Supplementary Table S2).

**TABLE 2:** Summary statistics of the datasets.

	FH train	FH test	OAK
Number of patients	121,644	15,075	1,187
Time [months] (median (95% CI))	19.33 (19.10–19.57)	19.83 (19.33–20.57)	11.43 (10.40–12.67)
Event = death (%)	72,068 (59.2)	8,875 (58.8)	854 (71.9)
Age at baseline [years] (mean (SD))	66.47 (10.98)	66.47 (11.05)	62.79 (9.57)
History of smoking [yes/no] (mean (SD))	0.84 (0.37)	0.84 (0.37)	0.83 (0.37)
Group stage (mean (SD))	3.31 (0.85)	3.31 (0.85)	3.43 (0.89)
ECOG value (mean (SD))	0.81 (0.80)	0.81 (0.80)	0.64 (0.49)
Neutrophils-lymphocytes ratio (NLR) [%] (mean (SD))	4.90 (4.86)	4.82 (4.66)	6.59 (6.31)
Body Mass index (BMI) [kg/m^2^] (mean (SD))	27.05 (5.96)	27.06 (5.92)	25.17 (4.80)
Number of metastasis sites (mean (SD))	0.37 (0.79)	0.36 (0.76)	1.46 (0.94)
Gender = male (%)	60,674 (49.9)	7,467 (49.5)	737 (62.1)
Alanine aminotransferase [enzymatic activity/volume] in serum or plasma [U/L] (mean (SD))	26.44 (29.47)	26.36 (29.24)	21.05 (13.80)
Calcium [mass/volume] in serum or plasma [mg/dL] (mean (SD))	9.33 (0.63)	9.33 (0.63)	9.40 (0.57)
Bilirubin total [mass/volume] in serum or plasma [mg/dL] (mean (SD))	0.57 (0.69)	0.56 (0.63)	0.47 (0.51)
Glucose [mass/volume] in serum or plasma [mg/dL] (mean (SD))	117.58 (34.19)	117.61 (34.35)	114.87 (33.02)
Protein [mass/volume] in serum or plasma [g/L] (mean (SD))	68.72 (7.16)	68.70 (7.18)	71.66 (6.61)
Urea nitrogen [mass/volume] in serum or plasma [mg/dL] (mean (SD))	17.87 (9.16)	17.81 (8.99)	26.37 (22.34)
Alkaline phosphatase [enzymatic activity/volume] in serum or plasma [U/L] (mean (SD))	114.71 (96.77)	114.57 (97.61)	118.84 (81.31)
Hemoglobin [mass/volume] in blood [g/dL] (mean (SD))	12.06 (1.97)	12.06 (1.96)	12.25 (1.67)
Chloride [moles/volume] in serum or plasma [mmol/L] (mean (SD))	101.17 (4.39)	101.14 (4.32)	101.18 (3.99)
Eosinophils/100 leukocytes in blood [%] (mean (SD))	2.54 (2.24)	2.55 (2.20)	2.59 (2.45)
Platelets [#/volume] in blood by automated count [10*9/L] (mean (SD))	264.80 (108.88)	265.96 (108.73)	281.13 (95.46)
Albumin [mass/volume] in serum or plasma [g/L] (mean (SD))	37.86 (5.39)	37.88 (5.38)	38.61 (5.70)
Lactate dehydrogenase [enzymatic activity/volume] in serum or plasma [U/L] (mean (SD))	278.18 (187.27)	276.51 (188.68)	295.28 (181.16)
Lymphocytes/100 leukocytes in blood by automated count [%] (mean (SD))	21.35 (13.11)	21.37 (13.14)	19.43 (9.43)
Monocytes [#/volume] in blood by automated count [10*9/L] (mean (SD))	0.68 (0.45)	0.68 (0.43)	0.65 (0.34)
Systolic blood pressure (mean (SD))	128.58 (19.36)	129.00 (19.19)	123.94 (16.92)
Heart rate (mean (SD))	83.18 (15.98)	83.25 (16.08)	84.38 (13.86)
Oxygen saturation in arterial blood by pulse oximetry [%] (mean (SD))	96.32 (2.39)	96.35 (2.35)	^a^
AST/ALT ratio [%] (mean (SD))	1.25 (0.63)	1.25 (0.63)	1.31 (0.61)

^a^ This covariate was not available in OAK.

To prepare the data for methods that require a full data matrix, all datasets were imputed with random forests by using the R package missForest (Stekhoven and Bühlmann, 2011). To prevent leakage of information between train and test sets, the imputation (random forest) was trained only on the train sets, and then applied to the FH test set and OAK test set.

### Models

One of our objectives in this paper was to determine if more complex survival models, that capture nonlinearities and feature dependence, are capable of predicting the patient’s risk better than the state of the art prognostic scores that are based on the classical Cox model. We selected the ROPRO (a Cox based model) as our baseline model and compared it against the regularized Cox model (Tibshirani 1997), random survival forest (Ishwaran et al., 2008), gradient boosting (Ridgeway, 1999), DeepSurv (Katzman et al., 2018) and a (to our knowledge) new autoencoder-based survival model (Goodfellow et al., 2016). In addition, we extended the super learner (van der Laan et al., 2007) framework to survival analysis problems and used it to aggregate the previous models into an ensemble that combined the predictions of all models, yielding a new weighted prediction.

Generally speaking, in survival analysis, the response variable is the time until an event occurs, such as death (Kalbfleisch and Prentice, 2002). If T represents a non-negative random variable that represents the time until the event, then the cumulative distribution function of T is called the survival function S. This function measures the probability of the event occurring after time t and is defined as

S(t) =P(T≥t), t ≥0.

The hazard function h is an alternative representation of the distribution of T. It represents the instantaneous rate of occurrence of the event at time t and is defined as

$$h\left(t\right)=\underset{dt\to 0}{lim}\frac{P\left(t\leq T<t\text{|}T\geq t\right)}{dt}.$$

The selected models in this paper all follow the underlying structure, but estimate the hazard function using different techniques.

### Multivariate Cox Model on Main Effects (ROPRO)

The ROPRO, introduced in (Becker et al., 2020), is a prognostic score based on the Cox model. The Cox model (Cox, 1972) is a widely used model in survival analysis that estimates the hazard function based on a set of given covariates of the population. It assumes that the hazard function h(t) is composed of two terms: a baseline hazard $h_{0}\left(t\right)$ that does not depend on the covariates and an exponential risk term $e^{r\left(X\right)}=e^{\beta X}$:

$$h\left(t\text{|}X\right)=h_{0}\left(t\right)\cdot e^{\beta X},$$

where X is the covariate vector and β are the model weights. The risk term integrates the interaction between the covariates and the hazard of each patient. In the case of the Cox model, the fitting focuses on the risk r(X) = βX, which is a linear function, using the following partial likelihood cost function:

$$log\;PL\left(\beta \right)=\overset{n}{\underset{i=1}{\sum }}\delta_{i}\left[r\left(X_{i}\right)-\log\left(\underset{l\in R\left(T_{i}\right)}{\sum }e^{r\left(x_{l}\right)}\right)\right]=\overset{n}{\underset{i=1}{\sum }}\delta_{i}\left[\beta X_{i}-\log\left(\underset{l\in R\left(T_{i}\right)}{\sum }e^{\beta X_{l}}\right)\right],$$

where $\delta_{i}$ is the censoring indicator. It is 1 if the patient has faced the event by the end of data collection and 0 otherwise. Naturally, being a linear function, it cannot implicitly deal with nonlinearities or interaction effects between the covariates (Harrell et al., 1996). This is one of the pitfalls of the Cox model and one of the reasons that motivated the creation of other more complex models (Ridgeway 1999; Katzman et al., 2018).

The authors of ROPRO started with a Cox model with 44 covariates and applied backward selection, removing the least significant covariates, until a total of 27 covariates remained in the model. The 27 selected covariates are represented in Table 2. In this work, we used the ROPRO formula as published in (Becker et al., 2020).

### Regularized Cox Model

The regularized Cox is a modification of the Cox proportional hazards algorithm where a regularization term is added to the cost function (Tibshirani 1997; Simon et al., 2011). The new regularized cost function has the form

$$log\;PL{\left(\beta \right)}_{RC}\;=\overset{n}{\underset{i=1}{\sum }}\delta_{i}\left[\beta X_{i}-\log\left(\underset{l\in R\left(T_{i}\right)}{\sum }e^{\beta X_{l}}\right)\right]+\lambda \left(\alpha \overset{p}{\underset{j=1}{\sum }}\left|\beta_{j}\right|+\frac{1}{2}\left(1-\alpha \right)\overset{p}{\underset{j=1}{\sum }}\beta_{j}^{2}\right).$$

The regularization term forces a penalization to the model weights β. The penalization depends on the type of regularization. The $L_{1}$ regularization (Lasso) performs covariate selection by setting some of the β values to 0, effectively removing them from the model (Tibshirani, 1997). $L_{2}$ regularization (ridge regression) scales the β values toward 0 but does not perform covariate selection, i.e. does not set the β to exactly 0. The elastic net combines $L_{1}$ and $L_{2}$. The parameter α determines which type of regularization is used, α=0 is the ridge regression, α=1 is Lasso, and values in between are the elastic net. Naturally, for values of α closer to 0 and 1, elastic net behaves more similar to ridge regression and Lasso, respectively.

### Gradient Boosting

Gradient boosting (GB) is a machine learning algorithm used in classification and regression problems (Friedman 2001). It builds the predictive model in an iterative fashion, in each iteration adding a weak learner that reflects the current residuals. By doing so, in each iteration the model should fit better to the data and consequently, reduce the prediction error.

GB can be applied to survival analysis by using the Cox partial likelihood (Cox 1972) as the cost function to determine the residuals (Ridgeway 1999). The new GB partial likelihood has the form

$$\text{log}PL{\left(\theta \right)}_{GB}=\overset{n}{\underset{i=1}{\sum }}\delta_{i}\left[{\hat{r}}_{GB}\left(X_{i}\right)-\log\left(\underset{l\in R\left(T_{i}\right)}{\sum }e^{{\hat{r}}_{GB\left(X_{l}\right)}}\right)\right].$$

Notice that the Cox model risk r(X) was substituted by ${\hat{r}}_{GB}\left(X\right)$, the predicting function fitted by GB. This predicting function is composed of multiple regression trees. Each of them fit on the residuals of the model of the previous iteration:

$${\hat{r}}_{GB}\left(X\right)=\overset{K}{\underset{k=1}{\sum }}\rho_{k}f_{k}\left(X\right),$$

where $f_{k}\left(x\right)$ corresponds to the model added in iteration k. As more models are added to the predictive model ${\hat{r}}_{GB}\left(x\right)$, the hazard function is estimated better (Ridgeway 1999).

### Random Survival Forest

Random survival forests (RSF) is a machine learning method that fits an ensemble of regression trees, a “forest”, that estimates the cumulative hazard function (Ishwaran et al., 2008). At each tree node, a covariate is used to separate the patients into groups. The RSF selects the split condition that maximizes the difference between the survival curves of the groups. Each tree is grown until it is not possible to create a new split that has more than a pre-specified number of unique events in each node.

### DeepSurv

DeepSurv uses a feed-forward neural network to predict the patient’s hazard h(t|X) (Katzman et al., 2018). It is composed of multiple fully connected layers that combine the covariates in a nonlinear way. In the final layer the predicted nonlinear risk function ${\hat{r}}_{DS}\left(t|x\right)$ is yielded. The loss function used to fit the model is based on the Cox partial hazard:

$$log\;PL{\left(\theta \right)}_{DS}\;=\overset{n}{\underset{i=1}{\sum }}\delta_{i}\left[{\hat{r}}_{DS}\left(t\text{|}X_{i}\right)-\log\left(\underset{l\in R\left(T_{i}\right)}{\sum }e^{{\hat{r}}_{DS\left(t\text{|}X_{l}\right)}}\right)\right]+\lambda {∥\beta ∥}_{2}^{2}.$$

### Autoencoder

Autoencoders (AE) are unsupervized neural networks composed of two components: 1) an encoder function that transforms the input X into an latent representation Z and 2) a decoder that transforms Z to $X_{reconstructed}$ (Goodfellow et al., 2016). The autoencoder is trained to minimize the difference between $X_{reconstructed}$ and X.

Here we exploit the autoencoder to perform dimensionality reduction. By setting Z to a lower dimensionality than X the autoencoder learns a representation that can best reconstruct X.

The autoencoder does not model the hazard function directly. Therefore, we use a Cox model to estimate the hazard from the intermediate representation. The new hazard is given by

$$h\left(t\text{|}Z\right)=h_{0}\left(t\right)\cdot e^{Z\beta }.$$

### Super Learner

Above we introduced multiple models that are capable of predicting the hazard function. All these algorithms have distinct structures, leading to different strengths and weaknesses in their estimation capability. The Super Learner (SL) (van der Laan et al., 2007) offers a framework to combine these models into a single model with the aim of combining the strengths and mitigating the weaknesses.

The SL was originally proposed to handle classification and regression problems. In this work we extend it to address time-to-event data and to use all the models described above.

Consider the dataset $O_{i}=\left(X_{i},T_{i},\delta_{i}\right)∼P_{0},\;i\;=\;1,...,\;n$ and the parameter of interest $\psi_{0}\left(X\right)$ which minimizes the cost function L(O,ψ) such that

$$\psi_{0}=arg\;min_{\psi \in \Psi }E_{0}L\left(O,\psi \right)$$

In this particular problem, $\psi_{0}\left(X\right)$ is a function that estimates the risk of a patient given its covariates. The SL framework uses *V*-fold cross-validation to split the dataset O into V distinct train-validation sets denoted by P(v) and V(v), respectively.

We learn a hazard function ${\hat{h}}_{k,v}$ from a given model k (e.g. DeepSurv) and a given training set P(v), and further test that model on the validation set V(v) to acquire predictions for each patient in V(v). Repeating this process for all *v*-s, the predicted hazards of model k are concatenated to form ${\hat{h}}_{k}$. This process is repeated for all k={1,...,11} models in our study. See Table 3 for the list of 11 models used to inform the SL model.

**TABLE 3:** Models used in the SL and their hyperparameters.

Model	Hyperparameters	Observations
ROPRO	—	
RSF	N = 500	
Regularized cox	α = 0	Lasso
	α = 0.25	Elastic net
	α = 0.5	
	α = 0.75	
	α = 1	Ridge regression
GB	N = 100; L = 1	
	N = 100; L = 2	
	N = 500; L = 1	
	N = 500; L = 2	
	N = 1,000; L = 1	
	N = 1,000; L = 2	
DS	Activation = *tanh*	All DS models had 1 hidden layer and 90 neurons in that hidden layer
	Activation = SELU	
AE	N = 1; *p* = 8	All AE models had *RELU* and sigmoid activation functions in the encoder and decoder parts
	N = 1; *p* = 14	
	N = 3; *p* = 8	
	N = 3; *p* = 14	

In the GB models, “N” and “L” correspond to the number of trees and their length, respectively. The “Activation” in the DS models corresponds to activation function used in the perceptrons, “N” corresponds to the number of hidden layers and “L” to the number of hidden neurons per layer. In the AE models, “N” corresponds to the number of layers of the encoder. “*p*” corresponds to the encoded variable size.

The next step in SL is to combine the predicted hazards of all k models to learn a new hazard function. This is done by using a linear model of the form

$${\hat{h}}_{SL}\left(t|x\right)=\overset{K}{\underset{k=1}{\sum }}\alpha_{k}\cdot {\hat{h}}_{k}\left(t|X\right),$$

where ${\hat{h}}_{SL}$ is the predicted SL hazard and $\alpha_{k}$ are the weights of the linear model. In the original SL, the weights $\alpha_{k}$ can be modeled in a variety of ways, e.g. Least Squares (van der Laan et al., 2007) or area under the curve (AUC) (LeDell et al., 2016). Our approach is based on (LeDell et al., 2016) but instead of using the AUC, we use the C-index (Harrell et al., 1982) as the objective function and maximize it using the L-BFGS-B algorithm (Byrd et al., 1995).

### Hyperparameter Tuning

As listed in Table 3, the more complex models require hyperparameters that adjust their complexity. Depending on the model, these hyperparameters were tuned by either cross-validation (for the regularized Cox models) or grid-search (for GB, RSF, DS and AE) on the FH training set.

### Model Testing

All models were fit using the training set and tested using the two distinct testing sets: FH in-sample test and OAK out-of-sample test set. The ROPRO model was taken pre-trained from the formula published in (Becker et al., 2020) and was not trained again, however it was tested equally against our test sets.

To assess the discrimination performance of the models, we used Harrell’s C-index (C-index) (Harrell et al., 1982). The C-index is a generalization of the AUC. It is a goodness of fit measure for survival models and measures the concordance between the risk/hazard values given by the model and the time-to-event. More specifically, it measures if patients that died earlier in time have a higher risk score than patients that died later. The statistic is defined from 0 to 1. Where 1 means perfect concordance, 0.5 means that the model is equivalent to a random guess and 0 represents perfect discordance. The C-index 95% confidence intervals (CI) were determined by bootstrapping. We use the confidence intervals to determine if one model has a significantly higher C-index than the other. In essence, this process is a comparison of two means, where the null hypothesis is $H_{0}:{\text{Cindex}}_{1}-{\text{Cindex}}_{2}=0$.

To further evaluate the discrimination of the models we used Uno’s C-index (Uno et al., 2011). Uno’s C-index is an extension of Harrell’s C-index that incorporates the censoring distribution into the score. This modification should make the C-index independent on the study’s censoring distribution (Uno et al., 2011).

### Sensitivity Analyses

Given the differences between the FH test set and OAK, we performed additional analyses to validate our results. The additional analyses include: 1) PCA analysis (Hastie et al., 2009) between FH test and OAK to verify differences between the datasets; 2) create an additional FH test set without the covariates not available in OAK and impute them to check the effect of these covariates. Further, 3) stratify the FH test set by cancer entity to check if C-index varies with cancer entity, and 4) permute FH train and test sets.

### Implementation

**FIGURE 1:**
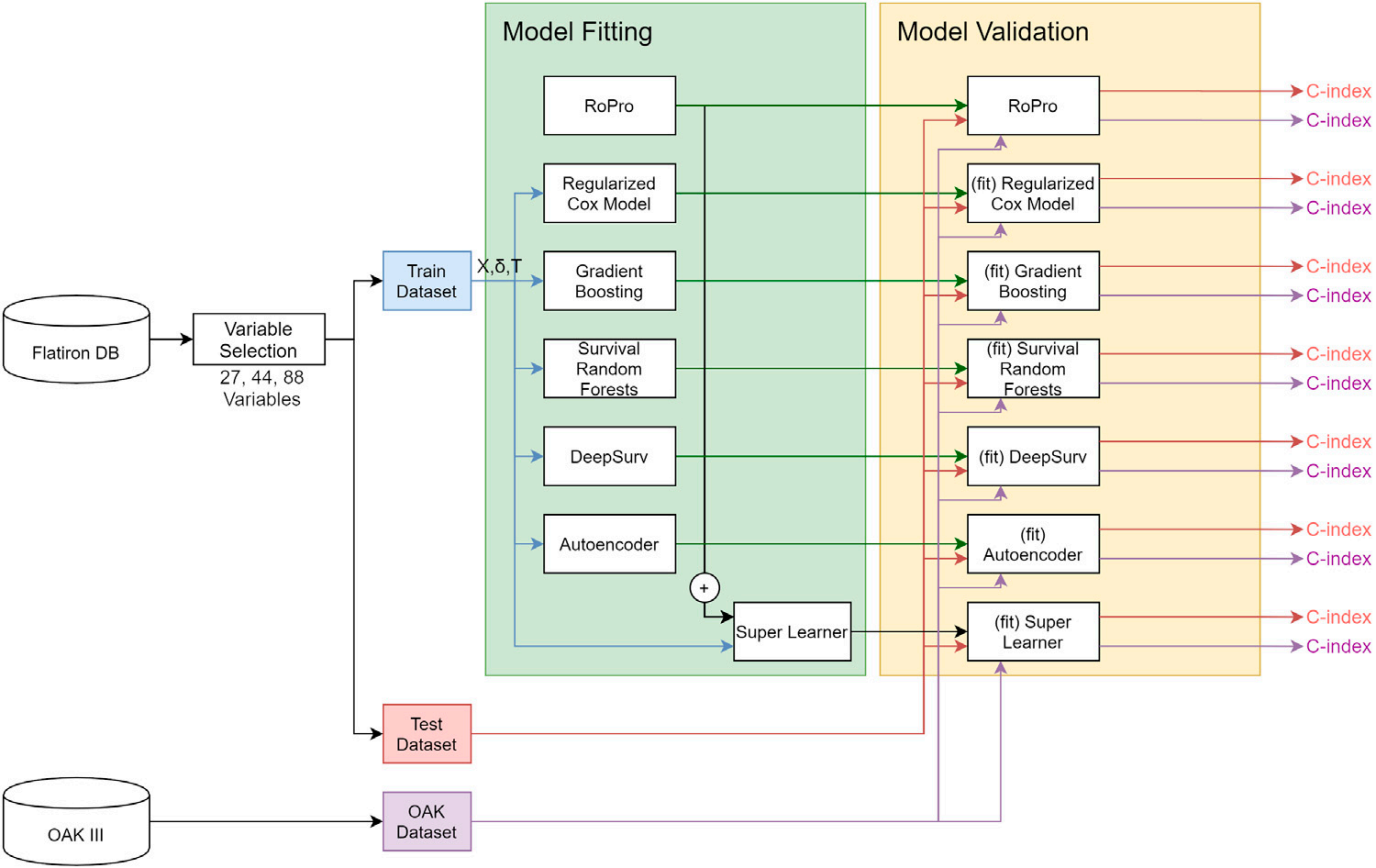
Diagram of the analysis.

All the analyses were done using R 3.5.1 (R Core Team 2018) and *Python* 3.6. The Cox model and C-index were used as implemented in the survival library (Therneau, 2015). The GB, RSF, and SL were used as implemented in the R libraries, gbm (Greenwell et al., 2019), RandomForestSRC (Ishwaran et al., 2008) and SuperLearner (Polley et al., 2019), respectively. DeepSurv was implemented in the *Python* DeepSurv package (Katzman et al., 2018). The random forest imputation was implemented in the missForest R library (Stekhoven and Bühlmann, 2011). The full analysis diagram is illustrated in Figure 1.

We modified the SuperLearner, DeepSurv and missForest packages to add new features used in this work. In the SuperLearner package, we added the functionality to process survival analysis problems. More specifically, we added new models (ROPRO, regularized Cox, RSF, GB, RF and AE) and a new fitting algorithm based on the C-index. In DeepSurv we added some functions to assess the quality of fit of the models. Finally, in the missForest package we added the functionality to save the fitted model and use it to impute new data, e.g. test sets that have to remain independent to the training. The modified packages and analysis files are available in the Supplementary materials.

## Results

**FIGURE 2:**
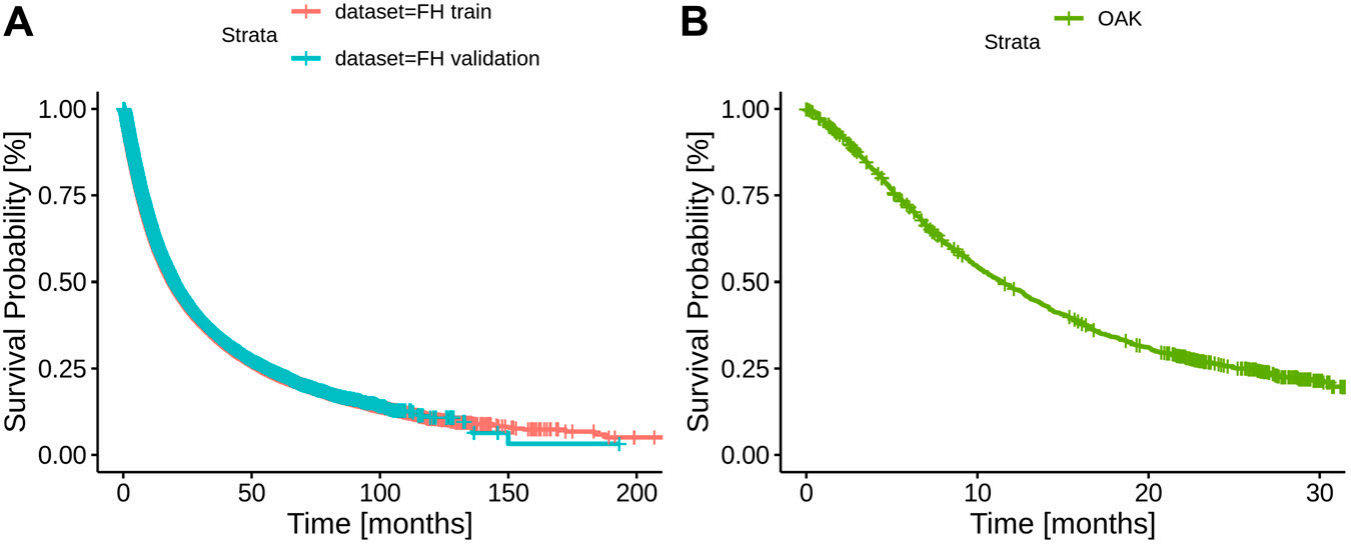
Kaplan Meier curves of the datasets. **(A)** - Kaplan Meier curves for the FH train and test datasets. **(B)** - Kaplan Meier curve for the OAK test set.

A total of three datasets were used in this analysis, FH train, FH test and OAK (see Methods) including cancer cohorts with a median follow-up time of 19.33 months (95% confidence interval (CI) 19.10–19.57), 19.83 (95% CI 19.33–20.57) and 11.43 (95% CI 10.40–12.67), respectively (Table 2; Figure 2).

Table 2 illustrates the summary statistics for the covariates in the 27 covariate feature set. The summary statistics for the 44 and 88 covariate feature sets are available Supplementary Table S2.

### Individual Model Development

We benchmarked the ROPRO against a set of eight more complex models - regularized cox with lasso, ridge regression and elastic net, GB, RSF, AE, DS and SL - across a total of three different feature sets, each with 27, 44 and 88 covariates yielding a total of 27 models.

**FIGURE 3:**
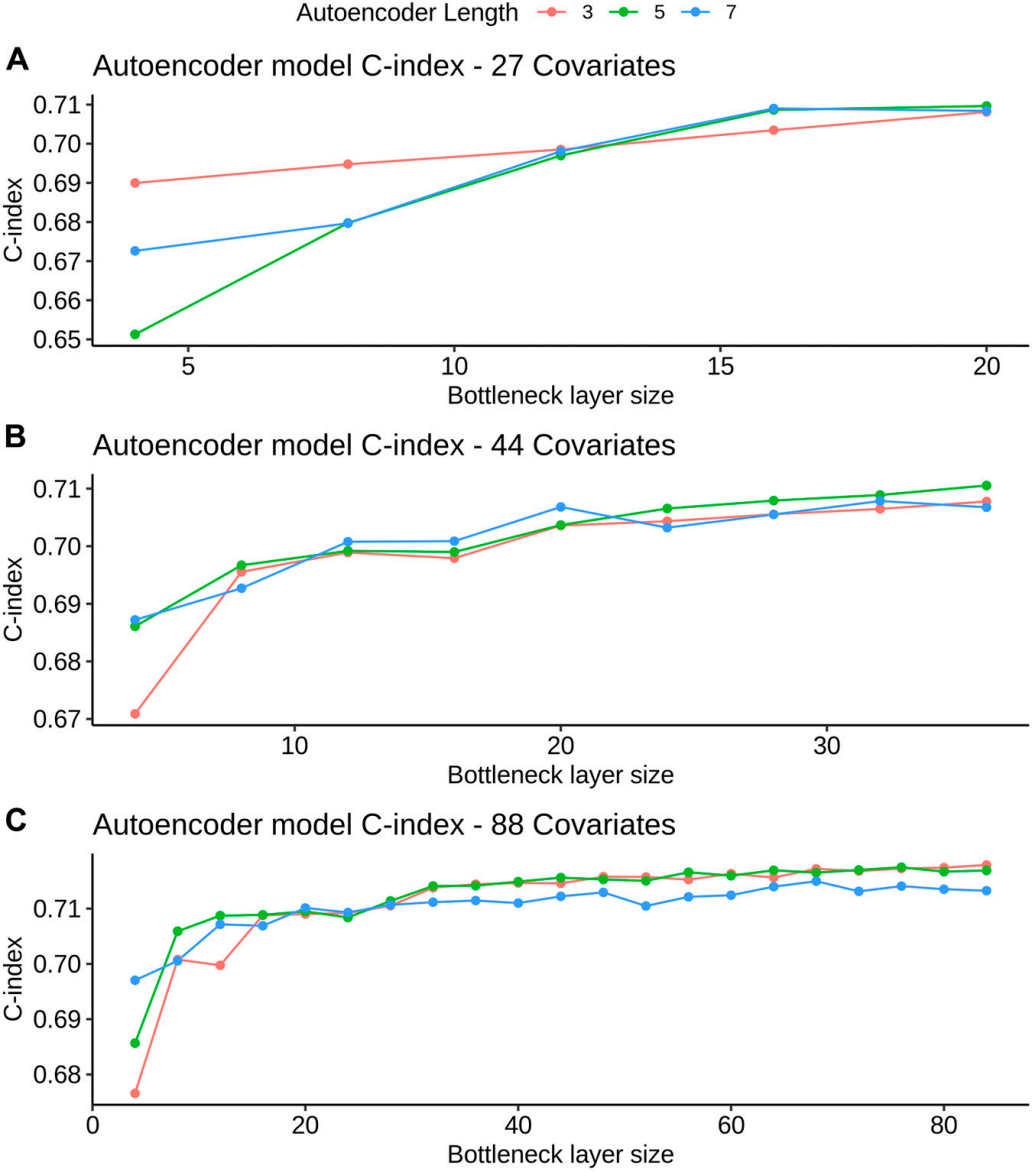
AE model C-index values for different bottleneck layer sizes and total layer sizes. All C-index values are referent to the validation set derived from FH train (see *Datasets* section for more details). Figures A, B and C refer to the 27, 44 and 88 covariate models, respectively.

After hyperparameter tuning (see Supplementary Table S3 for a list of tested hyperparameters), the optimal shrinkage in the regularized cox resulted in the selection by lasso and elastic net of 23, 27 and 49 covariates in the 27, 44 and 88 covariate models, respectively. With grid search, we determined that the optimal α value for the elastic net model was close to 1. To avoid having two lasso models, we fixed α=0.5. The optimal number of weak learners in GB and trees in RSF was 1,000. In DS, the optimal number of hidden layers and number of neurons in the hidden layer was (1 and 120), (1 and 150) and (1 and 180) for the 27, 44 and 88 covariate feature sets, respectively. An increase in hidden layer size did not lead to an improvement in DS performance, resulting in shallow models. The optimal activation function was the *SELU* for all feature sets. Lastly, in AE the C-index values for all the hyperparameter combinations are depicted in Figure 3. Overall, a higher bottleneck size resulted in a higher C-index value. The optimal bottleneck sizes were 20, 36 and 84 for the 27, 44 and 88 covariate sets. In terms of total number of layers, the optimal values were five layers for the 27 and 44 covariate sets and three for the 88 covariate set). We used *RELU* and sigmoid activation functions in the encoder and decoder parts, respectively.

**FIGURE 4:**
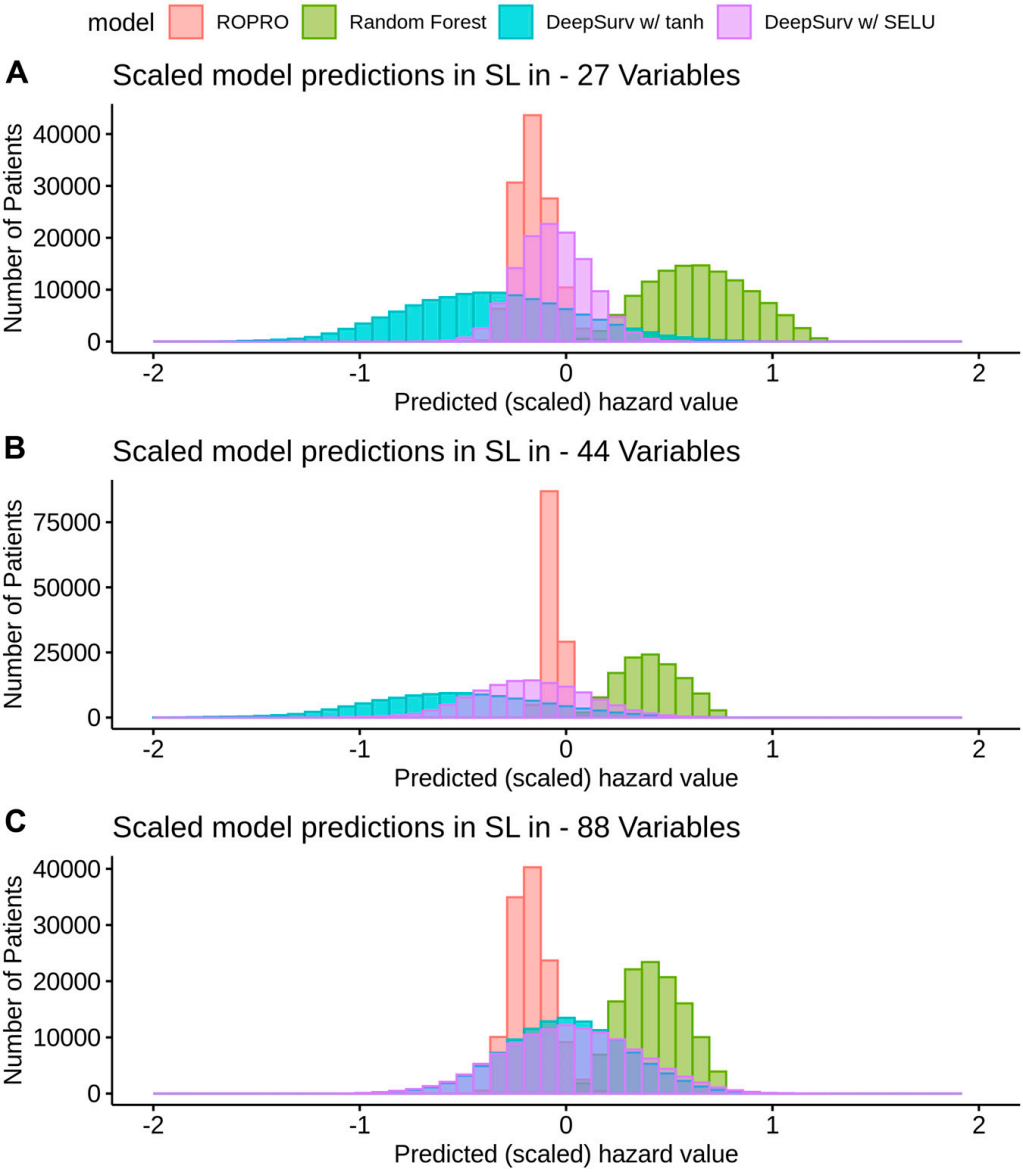
Histogram of the risk predictions for each model in the SL in the FH training dataset. The risk values correspond to the risk yielded by the original model, i.e., by ROPRO or DS. The risk was multiplied by the $\alpha_{k}$ value of the model. The $\alpha_{k}$ value scales the risks of each of the models in the SL. In the risk of the SL, only four models are represented, i.e. are not scaled down to zero. Those four models are both DS models, ROPRO and RSF.

**FIGURE 5:**
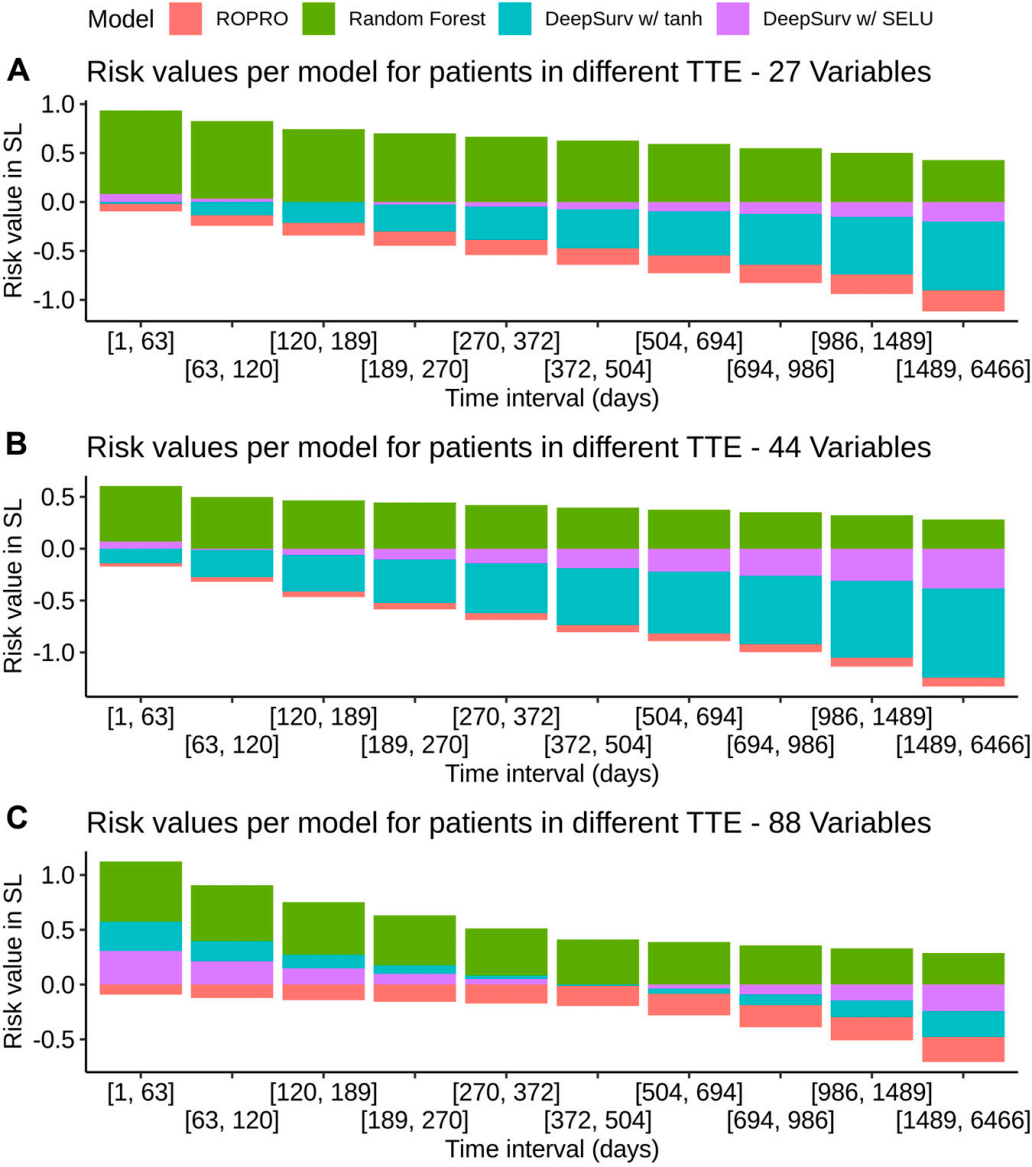
Individual model contribution to the SL risk by time-to-event in the FH training set. To create this visualization, the patients were split into groups based on their time to event (TTE). Each of these groups is represented in the *x*-axis. Then, for each group the median risk value per model was calculated and is displayed on the *y*-axis. The contribution changes over time because the models correctly assign higher risk for lower times-to-event and lower risk for later times-to-event.

K-fold cross-validation was used in the SL to calculate the contribution of each model (listed in Table 3) to the final score. Results show that independent from the feature set (27, 44 or 88 covariates) the only models that contributed to the SL score were the ROPRO, RSF and two versions of DS, one with tanh and another with *SELU* activation functions. Each model contributed to the SL distinctively (see Figure 4 for the models’ risk distributions). The models’ hazard value distributions varied for example, in center RSF (median 0.40–0.64) vs. ROPRO (median ‒0.161 to ‒0.0678), and in spread ROPRO (IQR 0.05–0.13) vs. DS with tanh (IQR 0.399–0.573). Additionally, the predicted risk values were stratified by the time-to-event of the patients (see Figure 5). In the 27 and 44 covariate models, RSF had the most sizable contribution for lower time-to-event (TTE). As the TTE increased, the contribution of RSF subsided while the contribution of both DS models increased. As a result, for later TTE, the model with the highest contribution changed from RSF to DS with *tanh*. Conversely, in the 88 covariate feature set, there was not a clear separation of the most contributive models.

### Model Performance

**FIGURE 6:**
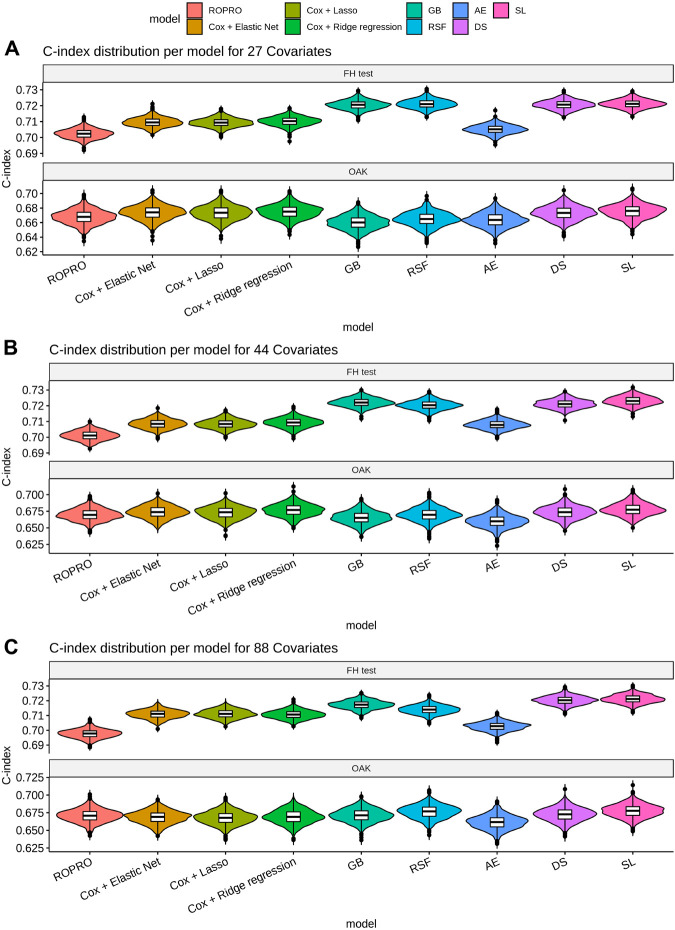
Violin plot of the C-index in the FH test dataset **(top)** and OAK III **(bottom)**. The C-index results for the 27, 44 and 88 covariate sets are illustrated in Figure **(A)**–**(C)**, respectively. The plot displays the distribution and a box-plot of the C-index. Bootstrap was used to determine the distribution of the C-index.

The C-index and corresponding 95% confidence intervals (CI) for the FH test dataset and the OAK test dataset are displayed in Figure 6 and Table 4, and Table 5. Figure 6 contains the C-index distributions for all models, datasets (FH test and OAK test) and feature sets (27, 44 and 88 covariates). Table 4 offers a more granular view of the C-index distributions from Figure 6, with information on each models’ C-index and 95% CI. Furthermore, Table 5 includes the Uno C-index and corresponding 95% CI.

**TABLE 4:** C-index and corresponding 95% confidence interval (CI) for all the models (ROPRO, regularized Cox models, Gradient Boosting (GB), Random Survival Forests (RSF), autoencoder (AE), DeepSurv (DS) and Super Learner (SL)) and covariate sets. Significant increases in C-index are in bold. Please refer to *Individual Model Development* section for the complete model hyperparameters.

#Covariates	Model	FH test		OAK	
		C-index	95% CI	C-index	95% CI
27 covariates	ROPRO^a^	0.702	[0.698, 0.707]	0.668	[0.652, 0.683]
	Cox + elastic net	0.709	[0.705, 0.714]	0.674	[0.657, 0.689]
	Cox + lasso	0.709	[0.705, 0.714]	0.674	[0.657, 0.690]
	Cox + ridge regression	0.710	[0.706, 0.715]	0.675	[0.659, 0.690]
	GB	**0.721**	**[0.716, 0.725]**	0.660	[0.644, 0.676]
	RSF	**0.721**	**[0.716, 0.726]**	0.665	[0.649, 0.680]
	AE	0.705	[0.700, 0.710]	0.664	[0.648, 0.680]
	DS	**0.721**	**[0.716, 0.725]**	0.673	[0.658, 0.689]
	SL	**0.721**	**[0.717, 0.726]**	0.676	[0.659, 0.691]
44 covariates	ROPRO^a^	0.701	[0.696, 0.706]	0.670	[0.657, 0.685]
	Cox + elastic net	0.708	[0.704, 0.714]	0.674	[0.658, 0.689]
	Cox + lasso	0.708	[0.704, 0.714]	0.674	[0.657, 0.687]
	Cox + ridge regression	0.709	[0.704, 0.714]	0.677	[0.661, 0.692]
	GB	**0.722**	**[0.718, 0.727]**	0.665	[0.650, 0.681]
	RSF	**0.720**	**[0.716, 0.725]**	0.670	[0.654, 0.686]
	AE	0.708	[0.703, 0.713]	0.660	[0.645, 0.676]
	DS	**0.721**	**[0.717, 0.726]**	0.674	[0.658, 0.689]
	SL	**0.723**	**[0.718, 0.728]**	0.677	[0.662, 0.695]
88 covariates	ROPRO^a^	0.698	[0.693, 0.702]	0.671	[0.656, 0.686]
	Cox + elastic net	**0.711**	**[0.706, 0.716]**	0.669	[0.653, 0.684]
	Cox + lasso	**0.711**	**[0.706, 0.716]**	0.668	[0.653, 0.683]
	Cox + ridge regression	**0.711**	**[0.706, 0.715]**	0.669	[0.652, 0.685]
	GB	**0.717**	**[0.712, 0.722]**	0.671	[0.656, 0.686]
	RSF	**0.714**	**[0.709, 0.719]**	0.677	[0.660, 0.692]
	AE	0.703	[0.698, 0.707]	0.662	[0.646, 0.678]
	DS	**0.720**	**[0.716, 0.725]**	0.673	[0.656, 0.688]
	SL	**0.721**	**[0.717, 0.726]**	0.678	[0.662, 0.692]

^a^ The ROPRO was applied to all feature sets (27, 44 and 88 covariates). In all feature sets the ROPRO only uses 27 covariates (it was not refit) but since each dataset was separately imputed, the C-index value changes between feature sets.

**TABLE 5:** Uno C-index and corresponding 95% confidence intervals. Significant increases in C-index over the ROPRO model are in bold.

#Covariates	Model	FH test		OAK	
		Uno C-index	95% CI	Uno C-index	95% CI
27 covariates	ROPRO	0.674	[0.669, 0.679]	0.653	[0.637, 0.668]
	Cox + elastic net	0.683	[0.678, 0.688]	0.659	[0.643, 0.674]
	Cox + lasso	0.683	[0.678, 0.688]	0.659	[0.643, 0.674]
	Cox + ridge regression	0.683	[0.678, 0.688]	0.660	[0.644, 0.675]
	GB	**0.695**	**[0.690, 0.700]**	0.645	[0.629, 0.661]
	RSF	**0.697**	**[0.692, 0.701]**	0.650	[0.635, 0.666]
	AE	0.679	[0.674, 0.684]	0.650	[0.634, 0.665]
	DS	**0.694**	**[0.689, 0.699]**	0.658	[0.644, 0.674]
	SL	**0.695**	**[0.690, 0.700]**	0.660	[0.645, 0.676]
44 covariates	ROPRO	0.672	[0.667, 0.676]	0.655	[0.640, 0.670]
	Cox + elastic net	0.680	[0.675, 0.686]	0.659	[0.644, 0.675]
	Cox + lasso	0.680	[0.675, 0.686]	0.659	[0.644, 0.673]
	Cox + ridge regression	0.681	[0.675, 0.686]	0.662	[0.647, 0.676]
	GB	**0.697**	**[0.691, 0.701]**	0.651	[0.635, 0.666]
	RSF	**0.693**	**[0.688, 0.698]**	0.655	[0.640, 0.670]
	AE	0.681	[0.675, 0.686]	0.647	[0.631, 0.662]
	DS	**0.693**	**[0.688, 0.699]**	0.659	[0.644, 0.674]
	SL	**0.695**	**[0.690, 0.701]**	0.665	[0.648, 0.679]
88 covariates	ROPRO	0.669	[0.664, 0.674]	0.655	[0.640, 0.671]
	Cox + elastic net	**0.684**	**[0.679, 0.689]**	0.654	[0.640, 0.670]
	Cox + lasso	**0.684**	**[0.679, 0.689]**	0.654	[0.639, 0.669]
	Cox + ridge regression	**0.684**	**[0.679, 0.689]**	0.655	[0.640, 0.670]
	GB	**0.692**	**[0.687, 0.697]**	0.658	[0.643, 0.674]
	RSF	**0.687**	**[0.682, 0.692]**	0.663	[0.648, 0.678]
	AE	0.677	[0.672, 0.682]	0.648	[0.633, 0.664]
	DS	**0.695**	**[0.690, 0.700]**	0.658	[0.643, 0.674]
	SL	**0.695**	**[0.690, 0.700]**	0.664	[0.648, 0.680]

### FH Test Set

As we observed similar patterns across all feature sets, we report here only results corresponding to the 44 covariate feature set. In the FH test dataset, the ROPRO achieved C-index values [95% CI] of 0.701 [0.696, 0.706]. In comparison, more complex models obtained slightly higher C-index values than ROPRO. Across all ML-derived models, the AE consistently yielded the lowest C-index values (0.708 [0.703, 0.713]), followed by lasso and elastic net (C-index 0.708 [0.704, 0.714]) and ridge regression (C-index 0.709 [0.704, 0.714]). The model performances improved using RSF (c-index 0.720 [0.716, 0.725]), GB (C-index 0.722 [0.718, 0.727]), DS (C-index 0.721 [0.717, 0.726]) and lastly, SL (C-index 0.723 [0.718, 0.728]). However, given their 95% CI only GB, RSF, DS and SL obtained significant increases in C-index for all feature sets when compared to ROPRO. As an exception, in the 88 covariates feature set, the regularized Cox models (C-index [95%CI] lasso and elastic net 0.711 [0.706, 0.716]; ridge regression 0.711 [0.706, 0.715]) also had significant increases in C-index when compared with ROPRO (C-index [95% CI] 0.698 [0.693, 0.702]). The increases in C-index for the remaining models were not significantly different.

All Uno C-index values were lower than the respective (Harrell) C-index. Regardless, the models that obtained significant (Harrell) C-index increases also had significant Uno C-index increases. For the 44 covariate feature set, the GB (Uno C-index [95% CI] 0.697 [0.691, 0.701]), RSF (Uno C-index [95% CI] 0.693 [0.688, 0.698]), DS (Uno C-index [95% CI] 0.693 [0.688, 0.699]) and SL (Uno C-index [95% CI] 0.695 [0.690, 0.701]) obtained significantly higher Uno C-index values than ROPRO (Uno C-index [95% CI] 0.672 [0.667, 0.676]). Additionally, in the 88 covariates feature set, the regularized Cox models also had significantly higher Uno C-index than ROPRO.

#### OAK Test

In OAK we observed similar patterns for the different feature sets. For easier reporting the following results similarly correspond to the 44 covariate feature set. The ROPRO resulted in a C-index value [95% CI] of 0.670 [0.657, 0.685]. In comparison, the model that yielded the highest C-index was SL 0.677 [0.662, 0.695]. Nevertheless, we observed that, contrary to the results in the FH test set, the confidence intervals between ROPRO and SL (and all the remaining models) overlapped, hence no statistically significant difference was found. Likewise, in the OAK dataset no model obtained a significantly higher Uno C-index than the ROPRO.

#### FH Test Set–Sensitivity Analyses

The PCA analysis with the first two principal components is shown in the Supplementary Figure S1. The C-index and 95% CI are displayed in Supplementary Tables S4–7 for FH test set without the covariates unavailable in OAK, FH test stratified by cancer entity, and FH train and test permutations, respectively.

The PCA analysis illustrated that the FH test distribution exhibited a higher variance than OAK, with the FH test set having a variance of (3.704, 2.137) while the OAK population exhibited a variance of (2.690, 1.421).

There were only minor changes in the C-index values between the original FH test set and the FH test set without the covariates not present in OAK. Ultimately, the same models, GB, RSF, DS, and SL, obtained significantly higher C-index values that ROPRO for all feature sets. Furthermore, in the 88 covariate feature set, the regularized Cox models obtained significant increases in C-index.

The C-index values showed a considerable variation between cancer entities. Most cancer entities had lower C-index values than the complete FH test (that had C-index values between 0.698 and 0.723). Only diffuse large B-cell lymphoma (C-index between 0.715 and 0.741), and follicular cancer (C-index between 0.771 and 0.788) had C-index values higher than the whole FH test dataset. Acute myeloid leukemia, breast cancer, gastric cancer, head and neck cancer, and metastatic breast cancer had the lowest discriminative power with C-index values close to 0.650. Advanced non-small cell lung cancer, the largest cohort in the FH test set, had C-index values between 0.673 and 0.687. In all cohort/feature set combinations, none of the more complex models obtained a significant increase in C-index against the ROPRO.

We reshuffled the original FH dataset twice, generating two extra sets of FH train and test. There were only minor changes in C-index between the results of the primary analysis (in the section above) and the results from these two extra sets. More specifically, in the FH test, GB (C-index [95% CI] 0.724 [0.718, 0.729]; 0.723 [0.718, 0.728]), RSF (C-index [95% CI] 0.722 [0.717, 0.728]; 0.720 [0.715, 0.725]), DS (C-index [95% CI] 0.724 [0.718, 0.730]; 0.723 [0.718, 0.728]), and SL (C-index [95% CI] 0.725 [0.720, 0.731]; 0.724 [0.719, 0.729]) obtained significant C-index increases when compared with ROPRO (C-index [95% CI] 0.701 [0.695, 0.707]; 0.701 [0.695, 0.707]). As above, the C-index values refer to the 44 covariate feature set although we observed similar patterns for all feature sets. We presented for each model two C-index and 95% CI, each of which refers to one set of new FH train and test sets. In the 88 covariate feature set of the two new sets of FH train and test, the regularized Cox models also obtained significant increases in C-index. The only deviation in C-index significance observed compared to the primary analysis was that the AE model had a significant increase in C-index against ROPRO in the 88 covariate feature set of one of the sensitivity analyses.

## Discussion

We conducted an extensive benchmarking study to investigate: 1) whether the predictive power of prognostic scores in oncology could be improved by replacing the Cox model with more complex machine learning models and 2) whether increasing the number of covariates from 27 model-selected to 44 and 88 would increase the models’ performance. To that end, we performed a comprehensive head-to-head comparison between a classic Cox model-based approach (ROPRO) and more complex ML-based survival models including two novel methods employing autoencoder and super learner algorithms. Overall, our analysis suggests that neither increasing the number of covariates nor using complex machine learning models increases the performance of prognostic scores in oncology. In part, this might be explained by the absence in baseline clinical data (like blood work data and patient/disease characteristics) of complex covariate interactions that would have otherwise been learned by the more complex models. We hypothesize that the addition of rich patient/disease information in the form of imaging, genomics or longitudinal data could be the key to improving prognostic scores in cancer. These more complex data types, apart from adding prognostic factors to the models, should also contain information that are not easily extractable by classical methods (like the Cox model) which should lead to an increase in performance of the more complex models and therefore better prognostic performance.

To our knowledge, this is to date the largest benchmarking study of prognostic scores in oncology both in terms of number of models and patients. Previous analyses that compared the performance of simple and complex machine learning models have yielded rather inconsistent results. Some of these studies have demonstrated improvements in using complex models against the classic Cox model. For instance, a recent study challenged the Cox model against random survival forests (RSF) and DeepSurv (DS) to derive prognostic scores among patients with oral cancer (Kim et al., 2019). The DS was overall the model with the highest C-index. Yet, the study was limited by the low number of 255 patients and nine covariates. A separate study applied the Cox model, RSF and regularized cox to a larger dataset comprising a population of 80,000 patients with cardiovascular disease (Steele et al., 2018). The authors concluded that the elastic net model (C-index 0.801) using 600 covariates performed better than the Cox model (C-index 0.793) using 27 covariates, but the overall improvement was only moderate. In comparison, three other studies did not find any noticeable improvements by employing machine learning models (Chen et al., 2019; Christodoulou et al., 2019; Desai et al., 2020). However, these studies compared the use of logistic regression with a binary endpoint against machine learning methods instead of the Cox model with a time-to-event endpoint. Our results showed that some of the more complex models, that could model covariate interactions and non-linear effects, did obtain significantly higher C-index values when compared to ROPRO. Although the C-index improvement size was still only moderate. Hence, the main results of the here presented study may contextualize the findings from (Chen et al., 2019; Christodoulou et al., 2019; Desai et al., 2020) to a survival analysis framework concluding that more complex machine learning models may not lead to a significant increase in performance over the Cox model.

Additionally, some of these studies also analyzed the effect of the covariate number in the prognostic score performance. The study by Kim et al. (Kim et al., 2019) analyzed models employing a range of five–nine covariates and found that the model performance increased with an increase in the covariate number. The same increase in performance was also observed when the performance of established prognostic scores (Arkenau et al., 2009; International Non-Hodgkin’s Lymphoma Prognostic Factors Project 1993; Ko et al., 2015; Kinoshita et al., 2013), which used a maximum of six covariates, was compared to the more recently developed ROPRO (Becker et al., 2020) that reported a number of 27 highly prognostic and independent covariates. Therefore, there is evidence that increasing the covariate size from small (less than 10 covariates) to a larger, but still moderate, number (30 covariates) leads to an increase in the prognostic score performance. This finding is not unexpected as the addition of more covariates increases the chances that some of them contain prognostic information that could be used by the models to increase their performance. In our analysis, we built upon the progress made in (Becker et al., 2020) by increasing the feature set from 27 to 44 and 88 covariates. However, the addition of these extra covariates did not lead to an increase in performance of the models as in previous studies. This could be caused by multiple reasons: First, the higher missingness in the 44 and 88 feature sets could have led to an erroneous imputation of the covariates with high missingness. Second, the 27 covariates included had been previously selected as the most relevant in (Becker et al., 2020), hence any additional covariates could have lower prognostic value. Both reasons should contribute to the lack of performance improvement. Yet, since the regularized Cox models did incorporate additional covariates from the 44 and 88 feature sets it gives evidence that there was some prognostic value in them although they did not lead to an increase in performance.

Conversely, in (Steele et al., 2018) two datasets with differing covariate numbers were studied: an expert-selected covariate dataset with 27 covariates vs. a much larger dataset with 600 covariates. The results demonstrated that the best 600 covariate model (elastic net) obtained a slightly higher C-index value than the best 27 covariate model (Cox model). The elastic net model added covariates such as prescription of cardiovascular medication (that should indicate severe cardiovascular problems) and prescription of laxatives/home visits (that might indicate general frailty). All these covariates are possibly associated (proxies) with cardiovascular disease but were not identified by the experts as prognostic, which may explain the increase in performance of the elastic net model. This result illustrates the need to incorporate more diverse data into prognostic scores. As explained above, we followed a different approach in this analysis and instead focused on increasing the number of biomarkers (blood work/patient characteristics) from 27 model-selected to 44 and 88 feature sets. Our results showed that this addition did not result in an increase in performance. We hypothesized that, perhaps, we had exhausted the available information in the blood work/patient characteristics in the 27 covariate dataset and the covariates added in the 44 and 88 feature sets did not carry prognostic information. Therefore, these results might suggest that perhaps there is a hard limit on the predictive power of baseline blood work/patient characteristics. To further increase the performance it might be necessary to incorporate other types of covariates as suggested by Steele et al. (Steele et al., 2018) or data with increased richness, like images, genomics or longitudinal biomarkers. Although, we would suggest that further research in this area is still needed.

Furthermore, all models had a comparable internal performance (C-index 0.70–0.72 within the FH test and C-index 0.66–0.68 within OAK) while the performance between datasets, which may be an indicator for model generalizability, was less strong. Particularly when the same models were compared between datasets, the C-index differences were more apparent. Some models had a considerable loss in performance with a decrease in C-index between FH test and OAK as high as 0.060 for GB or 0.056 for RSF. The ROPRO showed the most stable performance between datasets with a C-index difference as low as 0.027. These results suggest that the slight gains in performance achieved by the more complex models in the FH test dataset are not generalizable to other datasets. We hypothesize that this could happen due to multiple reasons: First, differences in the cohort number between FH test and OAK could cause differences in the C-index as the model performance could depend on the type of cancer. Second, the lack of some of the covariates in OAK, e.g., blood oxygen or granulocytes, could lead to a decrease in performance in the OAK dataset. Third, since OAK is a clinical trial, it is likely that the patient population is more homogeneous than in FH. Therefore, more extreme values in highly prognostic covariates (e.g. ECOG > 1) should be inexistent or rare, making it harder for prognostic prediction. Additionally, the study start date was defined differently between FH (first day of first line of treatment) and OAK (first day of second or third lines of treatment). We investigated some of these hypotheses in the sensitivity analyses above. For the first hypothesis, we tested whether the models had different performance for different cohorts. The FH test set C-index for advanced non-small cell lung cancer (the only cohort in OAK) ranged between 0.68–0.69, which is closer to the C-index in OAK. For the second hypothesis, we removed the covariates inexistent in OAK from the FH test and imputed them. This had little effect on the C-index of the FH test, therefore, we discard the effect of the absent OAK covariates. For the third hypothesis, we performed a PCA analysis where we compared both datasets, which supported the hypothesis that OAK has less extreme values. Given the sensitivity analyses, we argue that a combination of the first and third hypotheses is more likely. The C-index for the advanced non-small cell cancer in FH test was closer to the OAK value. Additionally, the other differences introduced in the third hypothesis might further decrease the C-index in the OAK dataset. Furthermore, there could have also been some overfitting to the FH test set that caused the decrease in OAK. Unfortunately, the compared prognostic scores in literature utilized test sets from the same data-source as the training set which makes a valid comparison not feasible. We suggest that further studies should be performed to investigate the true cause of this effect.

In general, we argue that in order to develop better prognostic scores in oncology, rather than focusing on more complex models on the same dataset, we should focus on getting access to larger and optimally multimodal data describing the patients in more detail. In particular, adding data about tumor biology via rich data types, e.g., via imaging, genomics or longitudinal data might be more beneficial and could lead to improved clinical decision-making when using prognostic scores. Consequently, these rich data types should contain complex information that the classical models cannot interpret, in that case, the more complex models tested in this work should demonstrate increased performance. Another area for improvement is related to the response of patients to treatments. By combining the patients’ treatments with longitudinal data, e.g. biomarkers, it might be possible to model the disease progression, leading to models that could offer real-time decision-making support. Overall, there remains an unmet clinical need for precise survival prediction to enable improved toxicity monitoring, treatment selection and assessment of clinical trial eligibility and hence further work is required to improve prognostic scores in oncology.

## Conclusion

Prognostic scores are important clinical decision-making tools for treatment decisions, monitoring adverse events, and clinical trial eligibility. Our results show that complex machine learning-derived models did not improve prognostic scores in oncology compared to a classical Cox-based framework. We argue that further research should focus on the impact of adding other data types (e.g. imaging, genomics or longitudinal biomarkers) describing complementary features of disease biology. In these scenarios, complex machine learning architectures might still prove beneficial.

## Data Availability

The data analyzed in this study is subject to the following licenses/restrictions: The data presented is owned by either Flatiron Health Inc. (train and in-sample test datasets) or F. Hoffmann-la Roche LTD (test dataset). Access to Flatiron Health may be made available upon request, and are subject to a license agreement with Flatiron Health. Requests to access these datasets should be directed to DataAccess@flatiron.com.
