# Supplementary material for: Artificial Intelligence for Prognostic Scores in Oncology: a Benchmarking Study
Source: Front Artif Intell. 2021 Apr 16;4:625573. doi: 10.3389/frai.2021.625573 (PMC8086599; doi:10.3389/frai.2021.625573)
Supplement: Supplementary file 1 [file datasheet1.zip › missForest/vignettes/missForest_1.4.pdf]

Using the `missForest` Package

Daniel J. Stekhoven
stekhoven@stat.math.ethz.ch

Friday, 13th of May, 2011
Update: Version 1.4, 31.12.13

Contents

1 Introduction **1**
1.1 What is this document? (And what it isn't!) 1
1.2 The `missForest` algorithm 2
1.3 Installation 2

2 Missing value imputation with `missForest` **2**
2.1 Description of the data used 2
2.2 `missForest` in a nutshell 3
2.3 Additional output using `verbose` 4
2.4 Changing the number of iterations with `maxiter` 5
2.5 Speed and accuracy trade-off manipulating `mtry` and `ntree` 8
2.5.1 `ntree` 8
2.5.2 `mtry` 9
2.6 Use subsampling instead of bootstrapping by setting `replace` to `FALSE` 9
2.7 Imbalanced data, stratified sampling and focussed selection (`classwt`, `cutoff`, `strata`, `sampsize`) 10
2.8 Controlling terminal nodes w.r.t. `nodesize` and `maxnodes` 11
2.9 Testing the appropriateness by supplying `xtrue` 11
2.10 Parallel execution of `missForest` using `parallelize` 13

3 Concluding remarks **14**

1 Introduction

1.1 What is this document? (And what it isn't!)

This *package vignette* is an application focussed user guide for the R package `missForest`. The functionality is explained using a couple of real data examples. Argument selection with respect to feasibility and accuracy issues are discussed and illustrated using these real data sets. Do not be alarmed by the length of this document which is mainly due to some major R output included for illustrative reasons.

This document is *not* a theoretical primer for the fundamental approach of the `missForest` algorithm. It also does not contain any simulations or comparative studies with other imputation methods. For this information we point the interested reader to Stekhoven and Bühlmann [2012].

1.2 The missForest algorithm

missForest is a nonparametric imputation method for basically any kind of data. It can cope with mixed-type of variables, nonlinear relations, complex interactions and high dimensionality ($p \gg n$). It only requires the observation (i.e. the rows of the data frame supplied to the function) to be pairwise independent. The algorithm is based on random forest (Breiman [2001]) and is dependent on its R implementation randomForest by Andy Liaw and Matthew Wiener. Put simple (for those who have skipped the previous paragraph): for each variable missForest fits a random forest on the observed part and then predicts the missing part. The algorithm continues to repeat these two steps until a stopping criterion is met or the user specified maximum of iterations is reached. For further details see Stekhoven and Bühlmann [2012].

To understand the remainder of this user guide it is important to know that missForest is running iteratively, continuously updating the imputed matrix variable-wise, and is assessing its performance between iterations. This assessment is done by considering the difference(s) between the previous imputation result and the new imputation result. As soon as this difference (in case of one type of variable) or differences (in case of mixed-type of variables) increase the algorithm stops.

missForest provides the user with an estimate of the imputation error. This estimate is based on the out-of-bag (OOB) error estimate of random forest. Stekhoven and Bühlmann [2012] showed that this estimate produces an appropriate representation of the true imputation error.

1.3 Installation

The R package missForest is available from the Comprehensive R Archive Network (CRAN, http://cran.r-project.org/) and as such can be installed in the default way using the install.packages function:

```
> install.packages(missForest, dependencies = TRUE)
```

Make sure to include the dependencies = TRUE argument to install also the randomForest package unless it is already installed.

2 Missing value imputation with missForest

In this section we describe using the missForest function. We will shed light on all arguments which can or have to be supplied to the algorithm. Also, we will discuss how to make missForest faster or more accurate. Finally, an interpretation of the OOB imputation error estimates is given.

2.1 Description of the data used

Iris data This complete data set contains five variables of which one is categorical with three levels. It is contained in the R base and can be loaded directly by typing data(iris). The data were collected by Anderson [1935].

Oesophageal cancer data This complete data set comes from a case-control study of oesophageal cancer in Ile-et-Vilaine, France. It is contained in the R base and can be loaded directly by typing data(esoph). The data were collected by Breslow and Day [1980].

Musk data This data set describes the shapes of 92 molecules of which 47 are musks and 45 are non-musks. Since a molecule can have many conformations due to rotating bonds, there are $n = 476$ different conformations in the set. The classification into musk and non-musk molecules is removed. For further details see Frank and Asuncion [2010].

2.2 `missForest` in a nutshell

After you have properly installed `missForest` you can load the package in your R session:

```
> library(missForest)
```

We will load now the famous Iris data set and artificially remove 10% of the entries in the data completely at random using the `prodNA` function from the `missForest` package:

```
> data(iris)
> iris.mis <- prodNA(iris, noNA = 0.1)
> summary(iris.mis)

  Sepal.Length    Sepal.Width     Petal.Length    Petal.Width   
 Min.   :4.300   Min.   :2.000   Min.   :1.000   Min.   :0.100  
 1st Qu.:5.100   1st Qu.:2.800   1st Qu.:1.600   1st Qu.:0.300  
 Median :5.800   Median :3.000   Median :4.400   Median :1.300  
 Mean   :5.862   Mean   :3.068   Mean   :3.856   Mean   :1.221  
 3rd Qu.:6.400   3rd Qu.:3.325   3rd Qu.:5.100   3rd Qu.:1.800  
 Max.   :7.900   Max.   :4.400   Max.   :6.900   Max.   :2.500  
 NA's   :17      NA's   :14      NA's   :17      NA's   :14     
       Species  
 setosa    :46  
 versicolor:44  
 virginica :47  
 NA's      :13  
```

We can see that there is an evenly distributed amount of missing values over the variables in the data set. With *completely at random* we mean that the process of deleting entries is not influenced by the data or the data generating process.

The missing data is now imputed by simply handing it over to `missForest` :

```
> iris.imp <- missForest(iris.mis)

  missForest iteration 1 in progress...done!
  missForest iteration 2 in progress...done!
  missForest iteration 3 in progress...done!
  missForest iteration 4 in progress...done!
  missForest iteration 5 in progress...done!
  missForest iteration 6 in progress...done!
```

Except for the iteration numbering no additional print-out is given. The results are stored in the R object `iris.imp` which is a list. We can call upon the imputed data matrix by typing `iris.imp$ximp`. *Note: A common mistake is to use* `iris.imp` *instead of* `iris.imp$ximp` *for subsequent analyses.*

Additionally, `missForest` provides an OOB imputation error estimate which can be extracted using the same `$` notation as with the imputed data matrix:

```
> iris.imp$OOBerror

     NRMSE        PFC 
0.13881247 0.05109489 
```

As mentioned before the Iris data set contains two types of variables, continuous and categorical. This is why the OOB imputation error supplies two values for the result of the imputation (default setting). The first value is the normalized root mean squared error (NRMSE, see Oba et al. [2003]) for the continuous part of the imputed data set, e.g., `Sepal.Length`, `Sepal.Width`, `Petal.Length` and `Petal.Width`. The second value is the proportion of falsely classified entries (PFC) in the categorical part of the imputed data set, e.g., `Species`. In both cases good performance of `missForest` leads to a value close to 0 and bad performance to a value around 1.

If you are interested in assessing the reliability of the imputation for single variables, e.g., to decide which variables to use in a subsequent data analysis, `missForest` can return the OOB errors for each variable separately instead of aggregating over the whole data matrix. This can be done using the argument `variablewise = TRUE` when calling the `missForest` function.

```
> iris.imp <- missForest(iris.mis, variablewise = TRUE)

  missForest iteration 1 in progress...done!
  missForest iteration 2 in progress...done!
  missForest iteration 3 in progress...done!

> iris.imp$OOBerror

       MSE        MSE        MSE        MSE        PFC
0.11222168 0.08926045 0.07041096 0.03225138 0.04379562
```

We can see that the output has the same length as there are variables in the data. For each variable the resulting error and the type of error measure, i.e., mean squared error (MSE) or PFC, is returned. Note that we are not using the NRMSE here.

2.3 Additional output using `verbose`

In 2.2 the print-out of `missForest` showed only which iteration is taking place at the moment. Anyhow, if you are imputing a large data set or choose to use ridiculously large `mtry` and/or `ntree` arguments (see 2.5) you might be interested in getting additional information on how `missForest` is performing.

By setting the logical `verbose` argument to `TRUE` the print-out is extended threefold:

`estimated error(s)` The OOB imputation error estimate for the continuous and categorical parts of the imputed data set. *Note: If there is only one type of variable there will be only one value with the corresponding error measure.*

`difference(s)` The difference between the previous and the new imputed continuous and categorical parts of the data set. The difference for the set of continuous variables $\mathbf{N}$ in the data set is computed by

$$\frac{\sum_{j\in\mathbf{N}}(\mathbf{X}^{imp}_{new} - \mathbf{X}^{imp}_{old})^2}{\sum_{j\in\mathbf{N}}(\mathbf{X}^{imp}_{new})^2},$$

and for the set of categorical variables the difference corresponds to the PFC.

`time` The runtime of the iteration in seconds.

If we rerun the previous imputation of the Iris data [1] setting `verbose = TRUE` we get:

[1] Since random forest – as its name suggests – is using a random number generator (RNG) the result for two trials on the same missing data set will be different. To avoid this from happening in the given illustrative example we use the `set.seed` function before applying `missForest` on the `iris.mis` data set. This causes the RNG to be reset to the same state as before (where we invisibly called `set.seed(81)` already but did not want to trouble the concerned reader with technical details).

```
> set.seed(81)
> iris.imp <- missForest(iris.mis, verbose = TRUE)

  missForest iteration 1 in progress...done!
    estimated error(s): 0.1479513 0.05109489
    difference(s): 0.008076659 0.06666667
    time: 0.106 seconds

  missForest iteration 2 in progress...done!
    estimated error(s): 0.1392889 0.05839416
    difference(s): 4.024134e-05 0
    time: 0.103 seconds

  missForest iteration 3 in progress...done!
    estimated error(s): 0.1420013 0.05109489
    difference(s): 1.289057e-05 0
    time: 0.129 seconds

  missForest iteration 4 in progress...done!
    estimated error(s): 0.139603 0.05839416
    difference(s): 8.446745e-06 0
    time: 0.105 seconds

  missForest iteration 5 in progress...done!
    estimated error(s): 0.1388125 0.05109489
    difference(s): 7.764542e-06 0
    time: 0.129 seconds

  missForest iteration 6 in progress...done!
    estimated error(s): 0.1395982 0.05839416
    difference(s): 1.581189e-05 0
    time: 0.1 seconds
```

The above print-out shows that `missForest` needs four iterations to finish. If we check the final OOB imputation error estimate:

```
> iris.imp$OOBerror

     NRMSE        PFC
0.13881247 0.05109489
```

we can see that it used the result from the second last iteration, i.e., the third instead of the last one. This is because the stopping criterion was triggered and the fact that the differences increase indicate that the new imputation is probably a less accurate imputation than the previous one. However, we can also see that the *estimated* error(s) is lower for the last imputation than for the one before. But we will show later on that the true imputation error is lower for iteration 3 (the impatient reader can jump to section 2.9).

2.4 Changing the number of iterations with `maxiter`

Depending on the composition and structure of the data it is possible that `missForest` needs more than the typical four to five iterations (see 2.3) until the stopping criterion kicks in. From

an optimality point of view we do want `missForest` to stop due to the stopping criterion and not due to the limit of iterations. However, if the difference between iterations is seriously shrinking towards nought and the estimated error is in a stalemate the only way to keep computation time at a reasonable level is to limit the number of iterations using the argument `maxiter`.

We show this using the `esoph` data. First, we run `missForest` on a data set where we removed 5% of the entries at random:

```
> data(esoph)
> esoph.mis <- prodNA(esoph, 0.05)
> set.seed(96)
> esoph.imp <- missForest(esoph.mis, verbose = TRUE)

  missForest iteration 1 in progress...done!
    estimated error(s): 0.4881112 0.6855808
    difference(s): 0.03651086 0.02651515
    time: 0.045 seconds

  missForest iteration 2 in progress...done!
    estimated error(s): 0.3984032 0.6786856
    difference(s): 0.0152145 0.007575758
    time: 0.077 seconds

  missForest iteration 3 in progress...done!
    estimated error(s): 0.4082038 0.6893564
    difference(s): 0.0001224566 0.003787879
    time: 0.044 seconds

  missForest iteration 4 in progress...done!
    estimated error(s): 0.4240482 0.7018025
    difference(s): 6.13314e-05 0.007575758
    time: 0.043 seconds

  missForest iteration 5 in progress...done!
    estimated error(s): 0.4272389 0.6895957
    difference(s): 0.0004251608 0.003787879
    time: 0.048 seconds

  missForest iteration 6 in progress...done!
    estimated error(s): 0.4049897 0.6946679
    difference(s): 0.0001733372 0.003787879
    time: 0.041 seconds

  missForest iteration 7 in progress...done!
    estimated error(s): 0.431403 0.6980152
    difference(s): 0.0001220771 0
    time: 0.046 seconds

  missForest iteration 8 in progress...done!
    estimated error(s): 0.4339154 0.7062959
    difference(s): 7.232987e-05 0.007575758
    time: 0.046 seconds
```

```
  missForest iteration 9 in progress...done!
    estimated error(s): 0.4852404 0.7020417
    difference(s): 0.0002884579 0.01515152
    time: 0.042 seconds
```

We can see that it takes `missForest` nine iterations to come to a stop. The returned imputation result was reached in iteration 8 having estimated errors of 0.55 and 0.73 and differences of $3 \cdot 10^{-5}$ and 0. In iteration 6 the estimated errors are smaller (i.e. 0.53 and 0.70) and the differences are $1 \cdot 10^{-4}$ and $4 \cdot 10^{-3}$. So why is `missForest` not simply taking the sixth iteration and calls it a day? Because the difference in the continuous part of the data set is still reduced in each iteration up until iteration 9. This stopping strategy is on average (taking all possible data sets into account) quite good but can have its caveats at specific data sets. In the above case of the `esoph` data we can get the result of the sixth iteration by doing the following:

```
> set.seed(96)
> esoph.imp <- missForest(esoph.mis, verbose = TRUE, maxiter = 6)

  missForest iteration 1 in progress...done!
    estimated error(s): 0.4881112 0.6855808
    difference(s): 0.03651086 0.02651515
    time: 0.045 seconds

  missForest iteration 2 in progress...done!
    estimated error(s): 0.3984032 0.6786856
    difference(s): 0.0152145 0.007575758
    time: 0.047 seconds

  missForest iteration 3 in progress...done!
    estimated error(s): 0.4082038 0.6893564
    difference(s): 0.0001224566 0.003787879
    time: 0.045 seconds

  missForest iteration 4 in progress...done!
    estimated error(s): 0.4240482 0.7018025
    difference(s): 6.13314e-05 0.007575758
    time: 0.042 seconds

  missForest iteration 5 in progress...done!
    estimated error(s): 0.4272389 0.6895957
    difference(s): 0.0004251608 0.003787879
    time: 0.041 seconds

  missForest iteration 6 in progress...done!
    estimated error(s): 0.4049897 0.6946679
    difference(s): 0.0001733372 0.003787879
    time: 0.043 seconds
```

The returned result is now given by iteration 6. Quintessentially, there are two uses for the `maxiter` argument:

1. Controlling the run time in case of stagnating performance;
2. extract a preferred iteration result not supplied by the stopping criterion.

2.5 Speed and accuracy trade-off manipulating `mtry` and `ntree`

`missForest` grows in each iteration for each variable a random forest to impute the missing values. With a large number of variables p this can lead to computation times beyond today's perception of feasibility. There are two ways to speed up the imputation process of `missForest`:

1. Reducing the number of trees grown in each forest using the argument `ntree`;
2. reducing the number of variables randomly sampled at each split using the argument `mtry`.

It is imperative to know that reducing either of these numbers will probably result in reduced accuracy. This is why we speak of a speed and accuracy *trade-off*.

2.5.1 `ntree`

The effect of reducing `ntree` on the computation time is linear, e.g., halving `ntree` will half computation time for a single iteration. The default value in `missForest` is set to 100 which is fairly large. Smaller values in the tens can give appropriate results already. We show this using the Musk data:

```
> musk.mis <- prodNA(musk, 0.05)
> musk.imp <- missForest(musk.mis, verbose = TRUE, maxiter = 3)

  missForest iteration 1 in progress...done!
    estimated error(s): 0.1491825
    difference(s): 0.02383702
    time: 280.739 seconds

  missForest iteration 2 in progress...done!
    estimated error(s): 0.1367353
    difference(s): 0.0001208087
    time: 277.011 seconds

  missForest iteration 3 in progress...done!
    estimated error(s): 0.137418
    difference(s): 3.836082e-05
    time: 278.287 seconds
```

The computation time is about 14 minutes and we end up with an estimated NRMSE of 0.14. *Note: The response was removed from the Musk data, that is why there is only the estimated NRMSE and also only the difference for the continuous part of the data set.*

If we repeat the imputation using the `ntree` argument and setting it to 20 we get:

```
> musk.imp <- missForest(musk.mis, verbose = TRUE, maxiter = 3, ntree = 20)

  missForest iteration 1 in progress...done!
    estimated error(s): 0.1724939
    difference(s): 0.02383371
    time: 56.705 seconds

  missForest iteration 2 in progress...done!
    estimated error(s): 0.1576795
    difference(s): 0.0002417658
```

```
    time: 55.833 seconds

  missForest iteration 3 in progress...done!
    estimated error(s): 0.1591702
    difference(s): 0.0001966117
    time: 56.053 seconds
```

The computation time is now around 3 minutes which is approximately a fifth of the previous computation time using 100 trees (as a matter of fact, taking the floor values of the iteration times in seconds then the former imputation took *exactly* five times longer than the latter). The estimated NRMSE has increased to 0.16 – an increase of 14% compared to before. In some application this might seem as an unacceptable increase of imputation error. However, if the number of variables is large enough, e.g., in the thousands like in gene expression data, the amount of computation time saved will surpass the amount of imputation error increased.

2.5.2 `mtry`

The effect on computation time when changing `mtry` is not as straight forward as with `ntree`. It is however more pronounced in settings with high-dimensionality (e.g. $p \gg n$, where n is the number of observations) and complex structures. The default setting in `missForest` is $\sqrt{p}$. This choice qualifies for a quite nice trade-off between imputation error and computation time. Anyhow, certain data might demand different choices either putting a focus on better imputation error or better computation time. We leave this delicate choice to the user of these certain data sets.

2.6 Use subsampling instead of bootstrapping by setting `replace` to `FALSE`

Like in the original paper by Breiman [2001] `missForest` uses bootstrap samples to grow its trees on. Another possibility would be to use subsamples instead. Randomly selected observations are replaced in the data when bootstrapping is performed, i.e., a single observation can be selected several times. In subsampling these observations are not replaced and thus a single observation can only be selected once. If `replace=FALSE` then `sampsize` (controlling the size of the sample drawn from the data to grow a tree) is reduced from n to $0.632n$. This is because otherwise there would be no more OOB observations and an error prediction would be impossible. The number 0.632 is the expected proportion of observations selected when using bootstrapping, i.e., selecting with replacements n observations.

```
> set.seed(81)
> iris.imp.sub <- missForest(iris.mis, verbose = TRUE, replace = FALSE)

  missForest iteration 1 in progress...done!
    estimated error(s): 0.1467573 0.04379562
    difference(s): 0.008238889 0.06666667
    time: 0.088 seconds

  missForest iteration 2 in progress...done!
    estimated error(s): 0.1410004 0.05109489
    difference(s): 4.998269e-05 0
    time: 0.104 seconds

  missForest iteration 3 in progress...done!
    estimated error(s): 0.1387039 0.04379562
```

```
    difference(s): 1.621601e-05 0
    time: 0.078 seconds

  missForest iteration 4 in progress...done!
    estimated error(s): 0.138982 0.05839416
    difference(s): 8.075441e-06 0
    time: 0.076 seconds

  missForest iteration 5 in progress...done!
    estimated error(s): 0.1400877 0.05109489
    difference(s): 1.10806e-05 0
    time: 0.083 seconds
```

```
> iris.imp.sub$OOBerror
```

```
     NRMSE        PFC
0.13898204 0.05839416
```

We can see that there is no substantial improvement compared to bootstrapping in the previous example in section 2.3 for the `iris` data. However, in some cases subsampling can be superior to bootstrapping. Therefore, if time allows explore both strategies and settle for the better performing one.

2.7 Imbalanced data, stratified sampling and focussed selection (`classwt`, `cutoff`, `strata`, `sampsize`)

From version 1.3 on missForest offers the possibility to pass more arguments to the `randomForest` function at its core. These include:

`classwt` adding priors to the classes in categorical variables;

`cutoff` setting cutoffs for each class in categorical variables;

`strata` perform stratified sampling for categorical variables;

`sampsize` define size of samples drawn from a variable.

For each of these arguments the user has to generate a list containing the appropriate object for each variable at the corresponding list entry, i.e., the third entry of the list corresponds to the third variable in the data, etc. The first three arguments in the above list do only make sense when used with categorical variables. However, the generated list has to have an entry for each variable - include for continuous variables `NULL` (for `cutoff` use `1`). The `sampsize` argument can be used for both types of data. In case of continuous variables a single integer and in case of categorical variables a vector of the same length as there are classes in the variable.

```
> iris.sampsize <- list(12, 12, 12, 12, c(10, 15, 10))
> iris.imp.sampsize <- missForest(iris.mis, sampsize = iris.sampsize)
```

Note how we set the list entry for `sampsize` in case of the fifth variable `Species` to a vector with three entries. An example for the use of `cutoff` could be:

```
> iris.cutoff <- list(1, 1, 1, 1, c(0.3, 0.6, 0.1))
> iris.imp.cutoff <- missForest(iris.mis, cutoff = iris.cutoff)
```

we set the cutoff for setosa to 0.3, for versicolor to 0.6 and for virginica to 0.1 (*not that this would make any sense - it is simply to show how the arguments have to be generated*). Equivalently, using a NULL instead of 1 for the continuous variables the input for classwt looks as:

```
> iris.classwt <- list(NULL, NULL, NULL, NULL, c(10, 30, 20))
> iris.imp.classwt <- missForest(iris.mis, classwt = iris.classwt)
```

2.8 Controlling terminal nodes w.r.t. nodesize and maxnodes

We can control the structural tree growing process two fold:

- by setting the maximum number of terminal nodes in the tree;
- by defining the minimum number of observations in a terminal node.

The default setting for the maximum number of nodes is given by the maximum possible in the tree growing process, subject to the limits of nodesize, which in turn has the default setting of 1 for continuous and 5 for categorical variables. The maxnodes argument is simply specified by an integer. For nodesize the user needs to supply a vector of length 2 where the first entry corresponds to continuous and the second entry to categorical variables:

```
> iris.imp.term <- missForest(iris.mis, nodesize = c(3, 7))
```

In the above call to missForest we set the number of observations in terminal nodes to 3 for continuous variables and 7 for categorical variables. Especially, the maxnodes argument can have a strong effect on computation time.

2.9 Testing the appropriateness by supplying xtrue

Whenever imputing data with real missing values the question arises how good the imputation was. In missForest the estimated OOB imputation error gives a nice indication at what you have to expect. A wary user might want to make an additional assessment (or back the OOB estimate up) by performing cross-validation or – in the optimal case – testing missForest previously on complete data. For both cases missForest offers the xtrue argument which simply takes in the same data matrix as xmis but with no missing values present. The strategy for testing the performance is the same as shown in the previous examples using prodNA:

1. Generate a data matrix with missing values;
2. impute this artificially generated data matrix;
3. compare the complete and imputed data matrices.

The functions to use for this strategy are prodNA, missForest and mixError. Using again the Iris data this would look like:

```
> iris.mis <- prodNA(iris, noNA = 0.1)
> iris.imp <- missForest(iris.mis)

> iris.err <- mixError(iris.imp$ximp, iris.mis, iris)
> print(iris.err)

    NRMSE       PFC
0.1242977 0.0000000
```

Note: We want to point out once more that the user has to extract the imputed matrix from the `missForest` *output using the* `$` *list notation. Not doing so will generate the following error:*

```
> iris.err <- mixError(iris.imp, iris.mis, iris)

Error in mixError(iris.imp, iris.mis, iris) :
  Wrong input for 'xmis' - you probably forgot to point at the
  list element $ximp from the missForest output object.
```

We can simplify the above strategy by using `xtrue`. If combined with `verbose = TRUE` the user even gets additional information on the performance of `missForest` between iterations:

```
> iris.imp <- missForest(iris.mis, xtrue = iris, verbose = TRUE)

  missForest iteration 1 in progress...done!
    error(s): 0.1236063 0
    estimated error(s): 0.1479513 0.05109489
    difference(s): 0.008076659 0.06666667
    time: 0.106 seconds

  missForest iteration 2 in progress...done!
    error(s): 0.1239567 0
    estimated error(s): 0.1392889 0.05839416
    difference(s): 4.024134e-05 0
    time: 0.126 seconds

  missForest iteration 3 in progress...done!
    error(s): 0.1245083 0
    estimated error(s): 0.1420013 0.05109489
    difference(s): 1.289057e-05 0
    time: 0.099 seconds

  missForest iteration 4 in progress...done!
    error(s): 0.1261406 0
    estimated error(s): 0.139603 0.05839416
    difference(s): 8.446745e-06 0
    time: 0.103 seconds

  missForest iteration 5 in progress...done!
    error(s): 0.1242977 0
    estimated error(s): 0.1388125 0.05109489
    difference(s): 7.764542e-06 0
    time: 0.1 seconds

  missForest iteration 6 in progress...done!
    error(s): 0.1242075 0
    estimated error(s): 0.1395982 0.05839416
    difference(s): 1.581189e-05 0
    time: 0.101 seconds
```

Supplying `xtrue` adds the line `error(s)` to the `missForest` output. We can observe that the true imputation error really is lower for the second last iteration as mentioned in section 2.2.

Additionally, the output object (in the above example `iris.imp`) contains now a list element `error` which can be called directly:

```
> iris.imp$error

    NRMSE       PFC
0.1242977 0.0000000
```

2.10 Parallel execution of `missForest` using `parallelize`

The argument `parallelize` allows to run `missForest` on multiple cores in parallel to save computational time. The parallel computation is achieved using the packages `foreach` (Revolution Analytics and Weston [2013b]) and `itertools` (Weston and Wickham [2010]). There are two possible ways to parallelize the algorithm of `missForest` :

1. **Create random forest objects parallel.**
   In each random forest grown divide `ntree` in k parts, where k equals the number of available cores. Each core computes the $\frac{\text{ntree}}{k}$ trees and finally the results are combined. This parallelization is most useful, if the random forest objects take long to compute and not too many variables with missing values are in the data. There are no consequences on the theoretical aspects of `missForest` regarding this parallelization (see Stekhoven and Bühlmann [2012]).

2. **Compute multiple iterations of `missForest` parallel.**
   An iteration of `missForest` consists of growing a random forest on the observed parts of a variable and subsequently predicting the missing parts using this forest. Partitioning the variables containing missing values into subsets of size k, where k equals the number of available cores, allows for the parallel computation of k iterations. When each of the k iterations is finished the missing values in the first k variables are updated and the next block of k variables is started. This parallelization is most useful if the data consists of many variables and the random forest objects do not take long to compute[2]. However, the methodology of this procedure deviates from the original `missForest` algorithm, where the missing values were updated after each iteration. Now, the values are only updated after the separate computation of the k iterations. This seems to have no negative effect on the performance of `missForest` .

The use of parallel computing with R requires an appropriate parallel backend telling R where the cores are and how many of them. There are several packages available offering this functionality. Here, we give a short example taken from Revolution Analytics and Weston [2013a] on how this can be achieved using the package `doParallel`. It is recommended to carefully read the documentation of `doParallel` (especially the Section "Registering the `doParallel` parallel backend").

The following code is loading the `doParallel` package and registers a (default) parallel backend. The function `getDoParWorkers()` returns the number of available cores.

```
> require(doParallel)
> registerDoParallel()
> getDoParWorkers()

[1] 2

> foreach(i=1:3) %dopar% sqrt(i)
```

[2]This can be achieved artificially by setting `ntree` or `mtry` relatively low, see Section 2.5

```
[[1]]
[1] 1

[[2]]
[1] 1.414214

[[3]]
[1] 1.732051
```

If the `foreach` loop returns the above output the backend is registered and can be used by `missForest` .

If the data has neither many variables nor the random forests take very long to compute, but the need for parallel computing is given, we recommend to try both parallelizations and see which improves the performance best.

3 Concluding remarks

Imputation using `missForest` can be done very easily. The OOB imputation error estimate facilitates the interpretation of such imputation results. However, it should always be kept in mind that imputing data with missing values does not increase the information contained within this data. It is only a way to have completeness for further data analysis. Many methods of data analysis require complete observations. In such complete case analyses observations missing only a single entry will be completely removed from the data and therefore the information content is reduced. Imputing the data beforehand prevents this reduction. For further details on the effect of imputation on the subsequent data analysis we suggest the books of Schafer [1997] and Little and Rubin [1987].

Acknowledgments

We thank Steve Weston for the input on the parallel computation approach.

References

E. Anderson. The irises of the gaspe peninsula. *Bulletin of the American Iris Society*, 59:2–5, 1935.

L. Breiman. Random forests. *Machine learning*, 45(1):5–32, 2001. ISSN 0885-6125.

N. E. Breslow and N. E. Day. Statistical methods in cancer research. 1: The analysis of case-control studies. *IARC Lyon / Oxford University Press*, 1980.

A. Frank and A. Asuncion. UCI machine learning repository, 2010. URL `http://archive.ics.uci.edu/ml`.

R.J.A. Little and D.B. Rubin. *Statistical Analysis with Missing Data.* Wiley New York, 1987. ISBN 0-471-80254-9.

S. Oba, M. Sato, I. Takemasa, M. Monden, K. Matsubara, and S. Ishii. A Bayesian missing value estimation method for gene expression profile data. *Bioinformatics*, 19(16):2088–2096, 2003. ISSN 1367-4803.

Revolution Analytics and Steve Weston. *doParallel: Foreach parallel adaptor for the parallel package*, 2013a. URL `http://CRAN.R-project.org/package=doParallel`. R package version 1.0.6.

Revolution Analytics and Steve Weston. *foreach: Foreach looping construct for R*, 2013b. URL `http://CRAN.R-project.org/package=foreach`. R package version 1.4.1.

J.L. Schafer. *Analysis of Incomplete Multivariate Data*. Chapman & Hall, 1997. ISBN 0-412-04061-1.

D.J. Stekhoven and P. Bühlmann. MissForest - nonparametric missing value imputation for mixed-type data. *Bioinformatics*, 2012. doi: 10.1093/bioinformatics/btr597.

Steve Weston and Hadley Wickham. *itertools: Iterator Tools*, 2010. URL `http://CRAN.R-project.org/package=itertools`. R package version 0.1-1.
