# Supplementary material for: Artificial Intelligence for Prognostic Scores in Oncology: a Benchmarking Study
Source: Front Artif Intell. 2021 Apr 16;4:625573. doi: 10.3389/frai.2021.625573 (PMC8086599; doi:10.3389/frai.2021.625573)

Supplementary Material

# Supplementary Tables

Supplementary table 1: Covariates available in each dataset and their percentage available.

|  | **Training set (27 covariates)** | **Training set (44 covariates)** | **Training set (88 covariates)** | **Percentage Available [%]** |
| --- | --- | --- | --- | --- |
| Number of Patients | 121644 | 121644 | 121644 |  |
| Time [months] (median (95% CI)) | 19.33 (19.10-19.57) | 19.33 (19.10-19.57) | 19.33 (19.10-19.57) | 100.00 |
| Event = Death (%) | 72068 (59.2) | 72068 (59.2) | 72068 (59.2) | 100.00 |
| Age at Baseline [years] (mean (SD)) | 66.46 (10.98) | 66.46 (10.98) | 66.46 (10.98) | 100.00 |
| History of Smoking [yes/no] (mean (SD)) | 43474 (84.0) | 43474 (84.0) | 43474 (84.0) | 42.43 |
| Group Stage (mean (SD)) | 3.31 (0.85) | 3.31 (0.85) | 3.31 (0.85) | 80.09 |
| ECOG Value (mean (SD)) | 0.81 (0.80) | 0.81 (0.80) | 0.81 (0.80) | 53.12 |
| Neutrophils-Lymphocytes Ratio (NLR) [%] (mean (SD)) | 4.90 (4.86) | 4.90 (4.87) | 4.90 (4.86) | 60.59 |
| Body Mass Index (BMI) [kg/m^2] (mean (SD)) | 27.05 (5.96) | 27.05 (5.96) | 27.05 (5.96) | 92.25 |
| Number of Metastasis Sites (mean (SD)) | 0.37 (0.79) | 0.37 (0.79) | 0.37 (0.79) | 100.00 |
| Gender = Male (%) | 60674 (49.9) | 60674 (49.9) | 60674 (49.9) | 100.00 |
| Alanine aminotransferase [enzymatic activity/volume] in serum or plasma [U/L] (mean (SD)) | 26.44 (29.47) | 26.44 (29.53) | 26.44 (29.52) | 82.54 |
| Calcium [mass/volume] in serum or plasma [mg/dL] (mean (SD)) | 9.33 (0.63) | 9.33 (0.63) | 9.33 (0.63) | 82.52 |
| Bilirubin.total [mass/volume] in serum or plasma [mg/dL] (mean (SD)) | 0.57 (0.69) | 0.57 (0.69) | 0.57 (0.69) | 80.72 |
| Glucose [mass/volume] in serum or plasma [mg/dL] (mean (SD)) | 117.58 (34.19) | 117.57 (34.18) | 117.58 (34.19) | 79.48 |
| Protein [mass/volume] in serum or plasma [g/L] (mean (SD)) | 68.72 (7.16) | 68.72 (7.16) | 68.72 (7.16) | 82.13 |
| Urea nitrogen [mass/volume] in serum or plasma [mg/dL] (mean (SD)) | 17.87 (9.16) | 17.87 (9.16) | 17.87 (9.16) | 84.65 |
| Alkaline phosphatase [enzymatic activity/volume] in serum or plasma [U/L] (mean (SD)) | 114.71 (96.77) | 114.71 (96.78) | 114.71 (96.78) | 83.33 |
| Hemoglobin [mass/volume] in blood [g/dL] (mean (SD)) | 12.06 (1.97) | 12.06 (1.97) | 12.06 (1.97) | 88.59 |
| Chloride [moles/volume] in serum or plasma [mmol/L] (mean (SD)) | 101.17 (4.39) | 101.17 (4.39) | 101.17 (4.39) | 62.04 |
| Eosinophils/100 leukocytes in blood [%] (mean (SD)) | 2.54 (2.24) | 2.54 (2.24) | 2.54 (2.24) | 44.68 |
| Platelets [#/volume] in blood by automated count [10*9/L] (mean (SD)) | 264.80 (108.88) | 264.80 (108.85) | 264.82 (108.86) | 78.01 |
| Albumin [mass/volume] in serum or plasma [g/L] (mean (SD)) | 37.86 (5.39) | 37.86 (5.39) | 37.86 (5.39) | 81.12 |
| Lactate dehydrogenase [enzymatic activity/volume] in serum or plasma [U/L] (mean (SD)) | 278.18 (187.27) | 278.19 (187.33) | 278.18 (187.28) | 32.01 |
| Lymphocytes/100 leukocytes in blood by automated count [%] (mean (SD)) | 21.35 (13.11) | 21.35 (13.10) | 21.35 (13.11) | 80.55 |
| Monocytes [#/volume] in blood by automated count [10*9/L] (mean (SD)) | 0.68 (0.45) | 0.68 (0.45) | 0.68 (0.45) | 72.03 |
| Systolic blood pressure (mean (SD)) | 128.58 (19.36) | 128.58 (19.36) | 128.58 (19.36) | 88.84 |
| Heart rate (mean (SD)) | 83.18 (15.98) | 83.18 (15.98) | 83.18 (15.98) | 95.63 |
| Oxygen saturation in arterial blood by pulse oximetry [%] (mean (SD)) | 96.32 (2.39) | 96.32 (2.39) | 96.32 (2.39) | 37.94 |
| AST/ALT ratio (mean (SD)) | 1.25 (0.63) | 1.25 (0.63) | 1.25 (0.63) | 86.32 |
| Aspartate aminotransferase [enzymatic activity/volume] in serum or plasma [U/L] (mean (SD)) |  | 24.49 (13.88) | 24.49 (13.89) | 83.33 |
| Carbon dioxide, total [moles/volume] in serum or plasma [mmol/L] (mean (SD)) |  | 25.71 (3.39) | 25.71 (3.39) | 68.94 |
| Eosinophils [#/volume] in blood [10*9/L] (mean (SD)) |  | 0.17 (0.10) | 0.17 (0.10) | 45.14 |
| Potassium [moles/volume] in serum or plasma [mmol/L] (mean (SD)) |  | 4.26 (0.48) | 4.26 (0.48) | 70.13 |
| Sodium [moles/volume] in serum or plasma [mmol/L] (mean (SD)) |  | 138.45 (3.62) | 138.45 (3.62) | 70.21 |
| Creatinine [mass/volume] in serum or plasma [mg/dL] (mean (SD)) |  | 0.93 (0.32) | 0.93 (0.32) | 86.52 |
| Erythrocytes [#/volume] in blood [10*12/L] (mean (SD)) |  | 4.10 (0.67) | 4.10 (0.67) | 80.06 |
| Hematocrit [volume fraction] of blood by automated count [%] (mean (SD)) |  | 36.78 (5.66) | 36.78 (5.66) | 85.49 |
| Basophils [#/volume] in blood by automated count [10*9/L] (mean (SD)) |  | 0.07 (0.05) | 0.07 (0.05) | 58.03 |
| Basophils/100 leukocytes in blood by automated count [%] (mean (SD)) |  | 0.59 (0.40) | 0.59 (0.40) | 56.88 |
| Granulocytes/100 leukocytes in blood [%] (mean (SD)) |  | 67.08 (17.49) | 67.08 (17.49) | 40.83 |
| Leukocytes [#/volume] in blood by automated count [10*9/L] (mean (SD)) |  | 9.89 (9.61) | 9.89 (9.61) | 83.63 |
| Lymphocytes [#/volume] in blood by automated count [10*9/L] (mean (SD)) |  | 2.12 (4.15) | 2.12 (4.14) | 80.19 |
| Monocytes/100 leukocytes in blood by automated count [%] (mean (SD)) |  | 7.87 (3.36) | 7.87 (3.36) | 72.73 |
| Neutrophils [#/volume] in blood by automated count [10*9/L] (mean (SD)) |  | 5.58 (2.72) | 5.58 (2.72) | 63.59 |
| Neutrophils/100 leukocytes in blood by automated count [%] (mean (SD)) |  | 61.50 (16.84) | 61.49 (16.86) | 39.49 |
| Diastolic blood pressure (mean (SD)) |  | 74.20 (10.60) | 74.20 (10.60) | 88.85 |
| Magnesium [mass/volume] in serum or plasma [mg/dL] (mean (SD)) |  |  | 1.95 (0.26) | 19.11 |
| Bilirubin direct [mass/volume] in serum or plasma [mg/dL] (mean (SD)) |  |  | 0.19 (0.12) | 6.61 |
| Bilirubin indirect [mass/volume] in serum or plasma [mg/dL] (mean (SD)) |  |  | 0.33 (0.22) | 3.31 |
| Thyrotropin [units/volume] in serum or plasma [iU/mL] (mean (SD)) |  |  | 0.00 (0.00) | 12.17 |
| Thyroxine (t4) free [mass/volume] in serum or plasma [ug/dL] (mean (SD)) |  |  | 0.00 (0.00) | 3.6 |
| Urate [mass/volume] in serum or plasma [mg/dL] (mean (SD)) |  |  | 5.35 (1.60) | 7.51 |
| Basophils/100 leukocytes in blood by manual count [%] (mean (SD)) |  |  | 1.28 (0.59) | 3.82 |
| Eosinophils [#/volume] in blood by manual count [10*9/L] (mean (SD)) |  |  | 0.20 (0.18) | 3.73 |
| Eosinophils/100 leukocytes in blood by manual count [%] (mean (SD)) |  |  | 2.40 (1.75) | 4.92 |
| Lymphocytes/100 leukocytes in blood by manual count [%] (mean (SD)) |  |  | 34.29 (29.94) | 5.93 |
| Monocytes/100 leukocytes in blood by manual count [%] (mean (SD)) |  |  | 5.76 (3.73) | 5.9 |
| Neutrophils [#/volume] in blood by manual count [10*9/L] (mean (SD)) |  |  | 4.28 (4.17) | 2.11 |
| Aptt in platelet poor plasma by coagulation assay [sec] (mean (SD)) |  |  | 28.50 (3.80) | 12.36 |
| Iga [mass/volume] in serum or plasma [g/L] (mean (SD)) |  |  | 1.34 (1.15) | 6.72 |
| Igg [mass/volume] in serum or plasma [g/L] (mean (SD)) |  |  | 10.02 (6.50) | 7.29 |
| Igm [mass/volume] in serum or plasma [g/L] (mean (SD)) |  |  | 0.50 (0.40) | 6.22 |
| Lambda light chains.free [mass/volume] in serum or plasma [mg/L] (mean (SD)) |  |  | 19.43 (19.30) | 4.39 |
| Kappa light chains.free [mass/volume] in serum [mg/L] (mean (SD)) |  |  | 64.82 (98.65) | 4.47 |
| Prothrombin time (pt) [sec] (mean (SD)) |  |  | 11.68 (1.65) | 16.24 |
| Ferritin [mass/volume] in serum or plasma [ng/mL] (mean (SD)) |  |  | 217.12 (209.76) | 16.21 |
| Inr in platelet poor plasma or blood by coagulation assay [ratio] (mean (SD)) |  |  | 1.07 (0.12) | 2.29 |
| Segmented neutrophils/100 leukocytes in blood [%] (mean (SD)) |  |  | 67.59 (13.12) | 2.29 |
| Thyroxine (t4) [mass/volume] in serum or plasma [ug/dL] (mean (SD)) |  |  | 5.82 (3.63) | 2.99 |
| C reactive protein [mass/volume] in serum or plasma [mg/L] (mean (SD)) |  |  | 17.03 (20.45) | 1.08 |
| Gamma glutamyl transferase [enzymatic activity/volume] in serum or plasma [U/L] (mean (SD)) |  |  | 32.21 (21.35) | 3.95 |
| Protein.monoclonal [mass/volume] in serum or plasma by electrophoresis [g/L] (mean (SD)) |  |  | 18.34 (16.20) | 1.45 |
| Bilirubin.conjugated [mass/volume] in serum or plasma [mg/dL] (mean (SD)) |  |  | 0.11 (0.11) | 2.97 |
| Band form neutrophils [#/volume] in blood [10*9/L] (mean (SD)) |  |  | 4.96 (2.89) | 6.26 |
| Creatinine [mass/volume] in urine [g/L] (mean (SD)) |  |  | 0.88 (0.57) | 1.51 |
| Protein [mass/volume] in urine [g/L] (mean (SD)) |  |  | 0.32 (0.34) | 1.77 |
| Blasts/100 leukocytes in blood by manual count [%] (mean (SD)) |  |  | 22.88 (26.40) | 1.23 |
| Segmented neutrophils/100 leukocytes in blood by manual count [%] (mean (SD)) |  |  | 49.94 (29.66) | 3.15 |
| Erythropoietin (epo) [units/volume] in serum or plasma [mIU/mL] (mean (SD)) |  |  | 34.68 (28.88) | 1.41 |
| Segmented neutrophils [#/volume] in blood [10*9/L] (mean (SD)) |  |  | 4.71 (3.29) | 1.29 |
| Monocytes [#/volume] in blood by manual count [10*9/L] (mean (SD)) |  |  | 0.57 (0.46) | 4.35 |
| Hemoglobin a1c/hemoglobin.total in blood [%] (mean (SD)) |  |  | 6.34 (1.07) | 1.19 |
| Protein [mass/time] in 24 hour urine [g/d] (mean (SD)) |  |  | 0.56 (0.68) | 1 |
| Erythrocyte sedimentation rate by westergren method [mm/hr] (mean (SD)) |  |  | 38.44 (32.28) | 2.59 |
| Band form neutrophils/100 leukocytes in blood by manual count [%] (mean (SD)) |  |  | 1.92 (2.20) | 4.59 |
| Granulocytes [#/volume] in blood [10*9/L] (mean (SD)) |  |  | 0.01 (0.00) | 36.85 |
| Body weight [kg] (mean (SD)) |  |  | 76.85 (18.45) | 97.8 |
| Pain severity - 0-10 verbal numeric rating [score] - reported (mean (SD)) |  |  | 0.48 (1.13) | 10.95 |
| Body surface area (mean (SD)) |  |  | 1.87 (0.25) | 1.94 |
| Respiratory rate (mean (SD)) |  |  | 18.10 (1.65) | 6.07 |

Supplementary table 2: Study population covariate values for the 88 covariate dataset.

|  | **FH Train** | **FH Test** | **OAK** |
| --- | --- | --- | --- |
| Number of Patients | 121644 | 15075 | 1187 |
| Time [months] (median (95% CI)) | 19.33 (19.10-19.57) | 19.83 (19.33-20.57) | 11.43 (10.40-12.67) |
| Event = Death (%) | 72068 (59.2) | 8875 (58.8) | 854 (71.9) |
| Age at Baseline [years] (mean (SD)) | 66.46 (10.98) | 66.47 (11.05) | 62.79 (9.57) |
| History of Smoking [yes/no] (mean (SD)) | 0.84 (0.37) | 0.84 (0.37) | 0.83 (0.37) |
| Group Stage (mean (SD)) | 3.31 (0.85) | 3.31 (0.85) | 3.43 (0.89) |
| ECOG Value (mean (SD)) | 0.81 (0.80) | 0.81 (0.80) | 0.64 (0.49) |
| Neutrophils-Lymphocytes Ratio (NLR) [%] (mean (SD)) | 4.90 (4.86) | 4.82 (4.66) | 6.59 (6.31) |
| Body Mass Index (BMI) [kg/m^2] (mean (SD)) | 27.05 (5.96) | 27.06 (5.92) | 25.17 (4.80) |
| Number of Metastasis Sites (mean (SD)) | 0.37 (0.79) | 0.36 (0.76) | 1.46 (0.94) |
| Gender = Male (%) | 60674 (49.9) | 7467 (49.5) | 737 (62.1) |
| Alanine aminotransferase [enzymatic activity/volume] in serum or plasma [U/L] (mean (SD)) | 26.44 (29.52) | 26.35 (29.21) | 21.05 (13.80) |
| Calcium [mass/volume] in serum or plasma [mg/dL] (mean (SD)) | 9.33 (0.63) | 9.33 (0.63) | 9.40 (0.57) |
| Aspartate aminotransferase [enzymatic activity/volume] in serum or plasma [U/L] (mean (SD)) | 24.49 (13.89) | 24.66 (14.17) | 23.32 (10.70) |
| Bilirubin total [mass/volume] in serum or plasma [mg/dL] (mean (SD)) | 0.57 (0.69) | 0.56 (0.62) | 0.47 (0.51) |
| Glucose [mass/volume] in serum or plasma [mg/dL] (mean (SD)) | 117.58 (34.19) | 117.59 (34.32) | 114.87 (33.02) |
| Protein [mass/volume] in serum or plasma [g/L] (mean (SD)) | 68.72 (7.16) | 68.70 (7.18) | 71.66 (6.61) |
| Urea nitrogen [mass/volume] in serum or plasma [mg/dL] (mean (SD)) | 17.87 (9.16) | 17.81 (8.99) | 26.37 (22.34) |
| Alkaline phosphatase [enzymatic activity/volume] in serum or plasma [U/L] (mean (SD)) | 114.71 (96.78) | 114.59 (97.65) | 118.84 (81.31) |
| Hemoglobin [mass/volume] in blood [g/dL] (mean (SD)) | 12.06 (1.97) | 12.06 (1.96) | 12.25 (1.67) |
| Magnesium [mass/volume] in serum or plasma [mg/dL] (mean (SD)) | 1.95 (0.26) | 1.94 (0.26) | 1.92 (0.26) |
| Bilirubin direct [mass/volume] in serum or plasma [mg/dL] (mean (SD)) | 0.19 (0.12) | 0.19 (0.12) | * |
| Bilirubin indirect [mass/volume] in serum or plasma [mg/dL] (mean (SD)) | 0.33 (0.22) | 0.32 (0.21) | * |
| Carbon dioxide, total [moles/volume] in serum or plasma [mmol/L] (mean (SD)) | 25.71 (3.39) | 25.73 (3.36) | * |
| Chloride [moles/volume] in serum or plasma [mmol/L] (mean (SD)) | 101.17 (4.39) | 101.13 (4.33) | 101.18 (3.99) |
| Eosinophils [#/volume] in blood [10*9/L] (mean (SD)) | 0.17 (0.10) | 0.18 (0.10) | 0.16 (0.10) |
| Eosinophils/100 leukocytes in blood [%] (mean (SD)) | 2.54 (2.24) | 2.55 (2.20) | 2.59 (2.45) |
| Thyrotropin [units/volume] in serum or plasma [iU/mL] (mean (SD)) | 0.00 (0.00) | 0.00 (0.00) | 0.00 (0.00) |
| Thyroxine (t4) free [mass/volume] in serum or plasma [ug/dL] (mean (SD)) | 0.00 (0.00) | 0.00 (0.00) | 0.00 (0.00) |
| Urate [mass/volume] in serum or plasma [mg/dL] (mean (SD)) | 5.35 (1.60) | 5.39 (1.64) | * |
| Basophils/100 leukocytes in blood by manual count [%] (mean (SD)) | 1.28 (0.59) | 1.34 (0.62) | 0.38 (0.32) |
| Eosinophils [#/volume] in blood by manual count [10*9/L] (mean (SD)) | 0.20 (0.18) | 0.20 (0.18) | 0.12 (0.09) |
| Eosinophils/100 leukocytes in blood by manual count [%] (mean (SD)) | 2.40 (1.75) | 2.41 (1.82) | 1.88 (1.53) |
| Lymphocytes/100 leukocytes in blood by manual count [%] (mean (SD)) | 34.29 (29.94) | 33.57 (29.23) | 19.17 (9.07) |
| Monocytes/100 leukocytes in blood by manual count [%] (mean (SD)) | 5.76 (3.73) | 5.90 (3.72) | 7.78 (3.05) |
| Neutrophils [#/volume] in blood by manual count [10*9/L] (mean (SD)) | 4.28 (4.17) | 4.45 (4.42) | 5.83 (2.53) |
| Aptt in platelet poor plasma by coagulation assay [sec] (mean (SD)) | 28.50 (3.80) | 28.44 (3.81) | 31.00 (4.63) |
| Iga [mass/volume] in serum or plasma [g/L] (mean (SD)) | 1.34 (1.15) | 1.34 (1.16) | * |
| Igg [mass/volume] in serum or plasma [g/L] (mean (SD)) | 10.02 (6.50) | 10.08 (6.30) | * |
| Igm [mass/volume] in serum or plasma [g/L] (mean (SD)) | 0.50 (0.40) | 0.51 (0.40) | * |
| Lambda light chains.free [mass/volume] in serum or plasma [mg/L] (mean (SD)) | 19.43 (19.30) | 19.76 (19.41) | * |
| Kappa light chains.free [mass/volume] in serum [mg/L] (mean (SD)) | 64.82 (98.65) | 62.58 (94.43) | * |
| Prothrombin time (pt) [sec] (mean (SD)) | 11.68 (1.65) | 11.65 (1.64) | * |
| Ferritin [mass/volume] in serum or plasma [ng/mL] (mean (SD)) | 217.12 (209.76) | 218.34 (210.09) | * |
| Inr in platelet poor plasma or blood by coagulation assay [ratio] (mean (SD)) | 1.07 (0.12) | 1.07 (0.12) | 1.03 (0.08) |
| Segmented neutrophils/100 leukocytes in blood [%] (mean (SD)) | 67.59 (13.12) | 67.34 (13.64) | * |
| Thyroxine (t4) [mass/volume] in serum or plasma [ug/dL] (mean (SD)) | 5.82 (3.63) | 5.75 (3.67) | * |
| C reactive protein [mass/volume] in serum or plasma [mg/L] (mean (SD)) | 17.03 (20.45) | 18.85 (20.73) | 20.42 (23.08) |
| Gamma glutamyl transferase [enzymatic activity/volume] in serum or plasma [U/L] (mean (SD)) | 32.21 (21.35) | 29.93 (20.09) | * |
| Protein.monoclonal [mass/volume] in serum or plasma by electrophoresis [g/L] (mean (SD)) | 18.34 (16.20) | 17.46 (15.63) | * |
| Bilirubin.conjugated [mass/volume] in serum or plasma [mg/dL] (mean (SD)) | 0.11 (0.11) | 0.11 (0.11) | * |
| Band form neutrophils [#/volume] in blood [10*9/L] (mean (SD)) | 4.96 (2.89) | 4.93 (2.87) | * |
| Creatinine [mass/volume] in urine [g/L] (mean (SD)) | 0.88 (0.57) | 0.89 (0.58) | * |
| Protein [mass/volume] in urine [g/L] (mean (SD)) | 0.32 (0.34) | 0.32 (0.33) | * |
| Blasts/100 leukocytes in blood by manual count [%] (mean (SD)) | 22.88 (26.40) | 23.51 (26.97) | * |
| Segmented neutrophils/100 leukocytes in blood by manual count [%] (mean (SD)) | 49.94 (29.66) | 49.16 (29.55) | * |
| Erythropoietin (epo) [units/volume] in serum or plasma [mIU/mL] (mean (SD)) | 34.68 (28.88) | 34.69 (29.48) | * |
| Segmented neutrophils [#/volume] in blood [10*9/L] (mean (SD)) | 4.71 (3.29) | 4.51 (3.26) | * |
| Monocytes [#/volume] in blood by manual count [10*9/L] (mean (SD)) | 0.57 (0.46) | 0.53 (0.45) | 0.60 (0.25) |
| Hemoglobin a1c/hemoglobin.total in blood [%] (mean (SD)) | 6.34 (1.07) | 6.51 (1.08) | * |
| Protein [mass/time] in 24 hour urine [g/d] (mean (SD)) | 0.56 (0.68) | 0.50 (0.59) | * |
| Platelets [#/volume] in blood by automated count [10*9/L] (mean (SD)) | 264.82 (108.86) | 265.96 (108.78) | 281.13 (95.46) |
| Potassium [moles/volume] in serum or plasma [mmol/L] (mean (SD)) | 4.26 (0.48) | 4.26 (0.48) | 4.28 (0.47) |
| Sodium [moles/volume] in serum or plasma [mmol/L] (mean (SD)) | 138.45 (3.62) | 138.46 (3.62) | 138.33 (3.20) |
| Erythrocyte sedimentation rate by westergren method [mm/hr] (mean (SD)) | 38.44 (32.28) | 36.33 (32.31) | * |
| Creatinine [mass/volume] in serum or plasma [mg/dL] (mean (SD)) | 0.93 (0.32) | 0.93 (0.31) | 0.91 (0.26) |
| Albumin [mass/volume] in serum or plasma [g/L] (mean (SD)) | 37.86 (5.39) | 37.89 (5.38) | 38.61 (5.70) |
| Lactate dehydrogenase [enzymatic activity/volume] in serum or plasma [U/L] (mean (SD)) | 278.18 (187.28) | 276.18 (187.97) | 295.28 (181.16) |
| Erythrocytes [#/volume] in blood [10*12/L] (mean (SD)) | 4.10 (0.67) | 4.11 (0.67) | 4.08 (0.56) |
| Hematocrit [volume fraction] of blood by automated count [%] (mean (SD)) | 36.78 (5.66) | 36.81 (5.64) | 37.15 (4.73) |
| Band form neutrophils/100 leukocytes in blood by manual count [%] (mean (SD)) | 1.92 (2.20) | 1.97 (2.21) | * |
| Basophils [#/volume] in blood by automated count [10*9/L] (mean (SD)) | 0.07 (0.05) | 0.07 (0.05) | 0.05 (0.04) |
| Basophils/100 leukocytes in blood by automated count [%] (mean (SD)) | 0.59 (0.40) | 0.60 (0.40) | 0.54 (0.37) |
| Granulocytes [#/volume] in blood [10*9/L] (mean (SD)) | 0.01 (0.00) | 0.01 (0.00) | * |
| Granulocytes/100 leukocytes in blood [%] (mean (SD)) | 67.08 (17.49) | 67.17 (17.51) | * |
| Leukocytes [#/volume] in blood by automated count [10*9/L] (mean (SD)) | 9.89 (9.61) | 9.82 (9.33) | 8.61 (4.15) |
| Lymphocytes [#/volume] in blood by automated count [10*9/L] (mean (SD)) | 2.12 (4.14) | 2.18 (4.34) | 1.35 (0.68) |
| Lymphocytes/100 leukocytes in blood by automated count [%] (mean (SD)) | 21.35 (13.11) | 21.37 (13.15) | 19.43 (9.43) |
| Monocytes [#/volume] in blood by automated count [10*9/L] (mean (SD)) | 0.68 (0.45) | 0.68 (0.43) | 0.65 (0.34) |
| Monocytes/100 leukocytes in blood by automated count [%] (mean (SD)) | 7.87 (3.36) | 7.86 (3.34) | 7.99 (3.35) |
| Neutrophils [#/volume] in blood by automated count [10*9/L] (mean (SD)) | 5.58 (2.72) | 5.54 (2.69) | 6.01 (2.75) |
| Neutrophils/100 leukocytes in blood by automated count [%] (mean (SD)) | 61.49 (16.86) | 61.48 (16.92) | 66.13 (8.63) |
| Body temperature (mean (SD)) | 97.87 (0.62) | 97.86 (0.61) | 97.60 (0.66) |
| Diastolic blood pressure (mean (SD)) | 74.20 (10.60) | 74.27 (10.56) | 73.33 (9.48) |
| Systolic blood pressure (mean (SD)) | 128.58 (19.36) | 129.00 (19.19) | 123.94 (16.92) |
| Heart rate (mean (SD)) | 83.18 (15.98) | 83.25 (16.08) | 84.38 (13.86) |
| Oxygen saturation in arterial blood by pulse oximetry [%] (mean (SD)) | 96.32 (2.39) | 96.35 (2.35) | * |
| Pain severity - 0-10 verbal numeric rating [score] - reported (mean (SD)) | 0.48 (1.13) | 0.48 (1.13) | * |
| Body surface area (mean (SD)) | 1.87 (0.25) | 1.87 (0.25) | 1.79 (0.22) |
| Respiratory rate (mean (SD)) | 18.10 (1.65) | 18.04 (1.60) | 17.22 (1.47) |
| AST/ALT ratio [%] (mean (SD)) | 1.25 (0.63) | 1.25 (0.63) | 1.31 (0.61) |

* - This variable is not available in the OAK dataset.

Supplementary table 3: Hyperparameter search space. In RSF, N_vars corresponds to the number of covariates

| **Model** | **Hyperparameters** | **Search space** |
| --- | --- | --- |
| **GB** | Number of trees | [100, 10000] |
|  | Tree length | [1, 3] |
| **RSF** | Number of trees | [100, 10000] |
|  | Number of variables tested for node | [N_vars/6, N_vars/2] |
|  | Number of data points in end node | [5, 25] |
|  | Number of splits to try in continuous covariates | [5, 25] |
| **DS** | Learning rate | [$10^{-7},10^{-3}]$ |
|  | Number or neurons per layer | [20, 150] |
|  | Number of layers | [1, 10] |
|  | Learning rate decay | [0, 0.001] |
|  | Momentum | [0.8, 0.95] |
|  | L1 regularization | [0, 0.5] |
|  | Activation function | SELU, tanh |
| **AE** | Learning rate | $10^{-3}$ |
|  | Number of layers | 3, 5, 7 |
|  | Bottleneck layer size | [4, N_vars-4] |
|  | Activation function | RELU (encoder), sigmoid (decoder) |

Supplementary table 4: C-index and 95% confidence intervals for the FH test set without the covariates not present in OAK removed and imputed in the same way as OAK. The supplementary table 2 contains all the covariates that were dropped from the FH dataset.

| **#Covariates** | **Model** | **FH Test** | |
| --- | --- | --- | --- |
|  |  | **C-index** | **95% CI** |
| **27 Covariates** | ROPRO | 0.703 | [0.698, 0.708] |
|  | Cox + Elastic Net | 0.710 | [0.706, 0.715] |
|  | Cox + Lasso | 0.710 | [0.705, 0.715] |
|  | Cox + Ridge regression | 0.711 | [0.706, 0.716] |
|  | GB | **0.720** | **[0.716, 0.725]** |
|  | RSF | **0.720** | **[0.716, 0.725]** |
|  | AE | 0.706 | [0.701, 0.711] |
|  | DS | **0.721** | **[0.716, 0.726]** |
|  | SL | **0.721** | **[0.717, 0.726]** |
| **44 Covariates** | ROPRO | 0.701 | [0.696, 0.705] |
|  | Cox + Elastic Net | 0.709 | [0.704, 0.714] |
|  | Cox + Lasso | 0.709 | [0.704, 0.713] |
|  | Cox + Ridge regression | 0.709 | [0.705, 0.714] |
|  | GB | **0.722** | **[0.717, 0.726]** |
|  | RSF | **0.720** | **[0.716, 0.725]** |
|  | AE | 0.707 | [0.702, 0.711] |
|  | DS | **0.720** | **[0.716, 0.725]** |
|  | SL | **0.723** | **[0.718, 0.727]** |
| **88 Covariates** | ROPRO | 0.698 | [0.693, 0.703] |
|  | Cox + Elastic Net | **0.711** | **[0.706, 0.716]** |
|  | Cox + Lasso | **0.711** | **[0.706, 0.715]** |
|  | Cox + Ridge regression | **0.710** | **[0.706, 0.715]** |
|  | GB | **0.716** | **[0.711, 0.721]** |
|  | RSF | **0.714** | **[0.709, 0.718]** |
|  | AE | 0.704 | [0.700, 0.709] |
|  | DS | **0.718** | **[0.714, 0.723]** |
|  | SL | **0.719** | **[0.714, 0.724]** |

Supplementary table 5: Harrell C-index and 95% confidence intervals for each cohort in the FH test set.

The table is present in a separate spreadsheet file.

Supplementary table 6: Harrell C-index and 95% confidence intervals for the **first** permuted (different) FH test set. (The C-index values in this table correspond to models fit with a different FH train set.) The new FH train and FH test sets were created by resampling (90% and 10%) from the complete FH dataset.

| **#Covariates** | **Model** | **FH Test** | | **OAK** | |
| --- | --- | --- | --- | --- | --- |
|  |  | **C-index** | **95% CI** | **C-index** | **95% CI** |
| **27 Covariates** | ROPRO | 0.702 | [0.697, 0.708] | 0.669 | [0.650, 0.688] |
|  | Cox + Elastic Net | 0.711 | [0.705, 0.717] | 0.675 | [0.657, 0.693] |
|  | Cox + Lasso | 0.711 | [0.706, 0.716] | 0.675 | [0.656, 0.694] |
|  | Cox + Ridge regression | 0.711 | [0.705, 0.716] | 0.675 | [0.656, 0.696] |
|  | GB | **0.722** | **[0.717, 0.728]** | 0.663 | [0.645, 0.681] |
|  | RSF | **0.723** | **[0.718, 0.729]** | 0.666 | [0.647, 0.686] |
|  | AE | 0.708 | [0.702, 0.714] | 0.656 | [0.638, 0.674] |
|  | DS | **0.722** | **[0.717, 0.727]** | 0.675 | [0.657, 0.696] |
|  | SL | **0.724** | **[0.718, 0.729]** | 0.679 | [0.658, 0.697] |
| **44 Covariates** | ROPRO | 0.701 | [0.695, 0.707] | 0.670 | [0.651, 0.688] |
|  | Cox + Elastic Net | 0.710 | [0.703, 0.715] | 0.676 | [0.658, 0.694] |
|  | Cox + Lasso | 0.709 | [0.704, 0.715] | 0.677 | [0.657, 0.696] |
|  | Cox + Ridge regression | 0.709 | [0.704, 0.715] | 0.677 | [0.659, 0.694] |
|  | GB | **0.724** | **[0.718, 0.729]** | 0.665 | [0.648, 0.684] |
|  | RSF | **0.722** | **[0.717, 0.728]** | 0.671 | [0.652, 0.688] |
|  | AE | 0.708 | [0.703, 0.714] | 0.667 | [0.648, 0.684] |
|  | DS | **0.724** | **[0.718, 0.730]** | 0.677 | [0.659, 0.695] |
|  | SL | **0.725** | **[0.720, 0.731]** | 0.678 | [0.659, 0.696] |
| **88 Covariates** | ROPRO | 0.703 | [0.697, 0.709] | 0.670 | [0.651, 0.689] |
|  | Cox + Elastic Net | **0.717** | **[0.712, 0.723]** | 0.670 | [0.652, 0.688] |
|  | Cox + Lasso | **0.717** | **[0.712, 0.723]** | 0.671 | [0.652, 0.691] |
|  | Cox + Ridge regression | **0.717** | **[0.712, 0.723]** | 0.672 | [0.652, 0.691] |
|  | GB | **0.726** | **[0.721, 0.732]** | 0.673 | [0.655, 0.691] |
|  | RSF | **0.719** | **[0.714, 0.724]** | 0.677 | [0.659, 0.696] |
|  | AE | **0.717** | **[0.711, 0.722]** | 0.667 | [0.648, 0.686] |
|  | DS | **0.728** | **[0.722, 0.734]** | 0.669 | [0.650, 0.688] |
|  | SL | **0.728** | **[0.723, 0.734]** | 0.679 | [0.659, 0.697] |

Supplementary table 7: Harrell C-index and 95% confidence intervals for the **second** permuted (different) FH test set. (The C-index values in this table correspond to models fit with another different FH train set.) The new FH train and FH test sets were created by resampling (90% and 10%) from the complete FH dataset.

| **#Covariates** | **Model** | **FH test** | | **OAK** | |
| --- | --- | --- | --- | --- | --- |
|  |  | **C-index** | **95% CI** | **C-index** | **95% CI** |
| **27 Covariates** | ROPRO | 0.703 | [0.698, 0.709] | 0.668 | [0.649, 0.688] |
|  | Cox + Elastic Net | 0.711 | [0.705, 0.717] | 0.675 | [0.655, 0.691] |
|  | Cox + Lasso | 0.711 | [0.705, 0.717] | 0.674 | [0.655, 0.692] |
|  | Cox + Ridge regression | 0.711 | [0.705, 0.717] | 0.674 | [0.655, 0.693] |
|  | GB | **0.722** | **[0.717, 0.727]** | 0.662 | [0.644, 0.680] |
|  | RSF | **0.722** | **[0.717, 0.728]** | 0.667 | [0.647, 0.685] |
|  | AE | 0.708 | [0.703, 0.714] | 0.659 | [0.640, 0.677] |
|  | DS | **0.722** | **[0.716, 0.727]** | 0.675 | [0.657, 0.694] |
|  | SL | **0.723** | **[0.718, 0.729]** | 0.676 | [0.657, 0.695] |
| **44 Covariates** | ROPRO | 0.701 | [0.695, 0.707] | 0.670 | [0.653, 0.688] |
|  | Cox + Elastic Net | 0.709 | [0.703, 0.715] | 0.677 | [0.656, 0.695] |
|  | Cox + Lasso | 0.709 | [0.703, 0.715] | 0.676 | [0.656, 0.693] |
|  | Cox + Ridge regression | 0.709 | [0.703, 0.714] | 0.677 | [0.659, 0.694] |
|  | GB | **0.723** | **[0.718, 0.728]** | 0.665 | [0.646, 0.684] |
|  | RSF | **0.720** | **[0.715, 0.725]** | 0.670 | [0.650, 0.687] |
|  | AE | 0.706 | [0.700, 0.711] | 0.667 | [0.649, 0.685] |
|  | DS | **0.723** | **[0.718, 0.728]** | 0.672 | [0.653, 0.693] |
|  | SL | **0.724** | **[0.719, 0.729]** | 0.677 | [0.659, 0.696] |
| **88 Covariates** | ROPRO | 0.702 | [0.696, 0.708] | 0.671 | [0.653, 0.690] |
|  | Cox + Elastic Net | **0.714** | **[0.708, 0.719]** | 0.671 | [0.653, 0.688] |
|  | Cox + Lasso | **0.714** | **[0.708, 0.719]** | 0.670 | [0.652, 0.689] |
|  | Cox + Ridge regression | **0.714** | **[0.708, 0.719]** | 0.671 | [0.653, 0.689] |
|  | GB | **0.724** | **[0.718, 0.729]** | 0.673 | [0.654, 0.693] |
|  | RSF | **0.716** | **[0.710, 0.721]** | 0.677 | [0.658, 0.695] |
|  | AE | 0.712 | [0.706, 0.718] | 0.660 | [0.640, 0.679] |
|  | DS | **0.724** | **[0.719, 0.730]** | 0.668 | [0.649, 0.687] |
|  | SL | **0.724** | **[0.719, 0.729]** | 0.682 | [0.663, 0.699] |

# Supplementary Figures

Supplementary figure 1: PCA analysis of the FH test and OAK datasets.


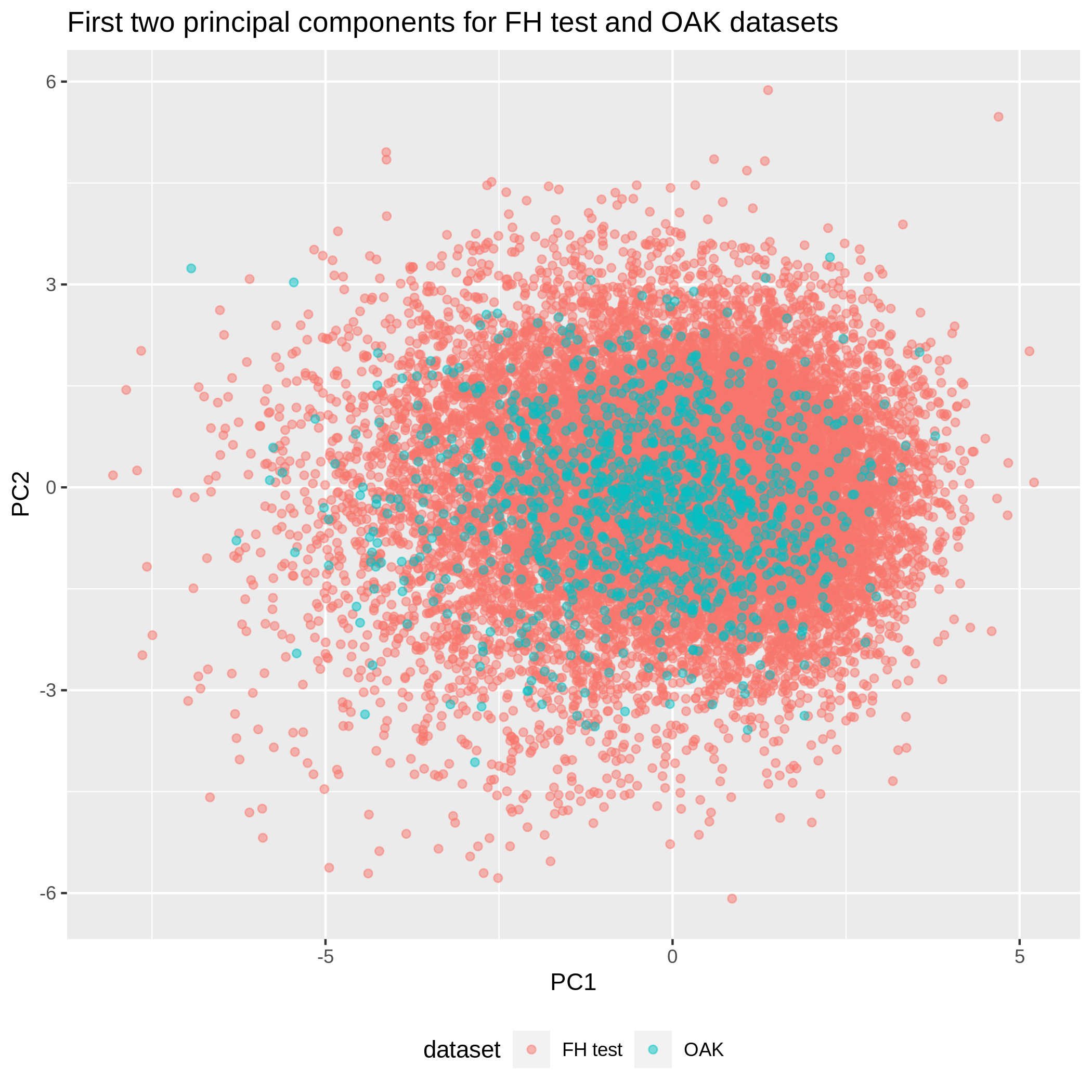

Supplement: Supplementary file 2 [file table1.docx]
