# Supplementary material for: Artificial Intelligence for Prognostic Scores in Oncology: a Benchmarking Study
Source: Front Artif Intell. 2021 Apr 16;4:625573. doi: 10.3389/frai.2021.625573 (PMC8086599; doi:10.3389/frai.2021.625573)
Supplement: Supplementary file 1 [file datasheet1.zip › SuperLearner/vignettes/SuperLearnerR.pdf]

Using the **SuperLearner** R Package

Eric Polley

Biometric Research Branch
National Cancer Institute
National Institute of Health

May 2011

Outline

1. SuperLearner
2. Boston Housing
3. ALL data
4. van't Veer data

The package is available at:
`https://github.com/ecpolley/SuperLearner`

These slides are available in the package and at:
`https://github.com/ecpolley/`

Need to install R packages **nnls** and **quadprog** before installing **SuperLearner**.

SuperLearner

Table: Main functions in the **SuperLearner** package

Function	Description
`SuperLearner`	fits super learner
`CV.SuperLearner`	cross-validate in super learner
`listWrappers`	returns list of wrappers in package
`write.SL.template`	prediction wrapper template
`write.screen.template`	screening wrapper template
`write.method.template`	method wrapper template

SuperLearner

```
fitSL <- SuperLearner(Y = Y,
                      X = X,
                      SL.library = c('SL.glm'),
                      family = gaussian(),
                      method = 'method.NNLS',
                      verbose = TRUE,
                      cvControl = list(V = 10))
```

Table: Main arguments for `SuperLearner`

Name	Description	Req.	Default
`Y`	outcome	Y	–
`X`	data.frame for fit	Y	–
`newX`	data.frame for predict	N	`X`
`SL.library`	library of algorithms	Y	–
`cvControl`	list for CV control	N	–
`control`	optional controls	N	–
`verbose`	detailed report	N	FALSE
`family`	error distribution	N	gaussian
`method`	loss function & model	N	NNLS
`id`	cluster id	N	–
`obsWeights`	observations weights	N	–

- `Y` and `X` are the data used to fit each algorithm (the learning data)
- `newX` is not required but can be a helpful shortcut.
  `newX` will not be used to fit the models.

Example with `X` and `newX`:

```
fit <- glm(Y ~ ., data = X)
out <- predict(fit, newdata = newX)
```

The formula `Y ~ .` means an additive linear model using all columns of `X`

`newX` might be a test set, the interesting values of `X` for prediction, a stacked data.frame with exposure levels set to be used with for G-computation, etc

Example setting exposure level for `newX`

```
newData <- rbind(
  cbind(A = 0, subset(X, select = -A)),
  cbind(A = 1, subset(X, select = -A))
  )
```

SuperLearner

- `family`: currently either `gaussian()` or `binomial()`.
- `method`: either ‘`method.NNLS`’, ‘`method.NNloglik`’, or your own method (see `create.method.template()`).
- `verbose`: helpful to set this to `TRUE` to see the progress of the estimation.

The ensemble model for "NNLS" is:

$$\Psi_{SL}(W) = \sum_{j=1}^{K} \alpha_j \Psi_j(W), \quad \alpha_j \geqslant 0, \sum \alpha_j = 1$$

The ensemble model for "NNloglik" is:

$$\Psi_{SL}(W) = \frac{1}{1 + \exp\left\{-\sum_{j=1}^{K} \alpha_j \operatorname{logit}_\gamma \left(\Psi_j(W)\right)\right\}}, \quad \alpha_j \geqslant 0, \sum \alpha_j = 1$$

where $\operatorname{logit}_\gamma$ is the trimmed logit function to control when $\Psi_j(W)$ is near 0 or 1.

There are two types of algorithms that can be used in `SL.library`:

1. Prediction algorithms. Algorithms that take as input X and Y and return a predicted Y value.
2. Screening algorithms. Algorithms designed to reduce the dimension of X. They take as input X and Y and return a logical vector indicating the columns in X passing the screening. Screening algorithms can be coupled with prediction algorithms to form new prediction algorithms.

`listWrappers()`

Show in R

```
listWrappers()
```

SuperLearner

There are two ways to specify the algorithms in `SL.library`:

1. A character vector:
   `c('SL.glm', 'SL.glmnet', 'SL.gam')`
2. A list of character vectors:
   `list(c('SL.glm', 'screen.corP'), 'SL.gam')`

If only using prediction algorithms, easier to use the first method.

If using screening algorithms, the list is required. The syntax for the elements in the list is the prediction algorithm is first, followed by the screening algorithms. Multiple screening algorithms can be used. If a singleton, the default is to apply to all variables.

see the help documents for `SuperLearner` for more examples of `SL.library`

Creating Wrappers

Many algorithms are included in the package (use `listWrappers()` for a list of included functions), but these are just enough to get you started.

A few reasons to build your own wrappers:

- Want to use an algorithm not currently included
- Problem suggests different values for the tuning parameters
- Want to include a range of tuning parameters, not just the default
- Want to select tuning parameters in a different way (e.g. `SL.glmnet` selecting λ)
- Force variables to be used in step-wise methods

Creating wrappers

The **SuperLearner** vignette contains a table of tuning parameters for the algorithms in the package

Show in R

```
vignette("SuperLearner")
```

Creating wrappers

Example: Creating new prediction algorithm wrapper

Creating wrappers

Consider the `polymars` algorithm in the **polspline** package.

- continuous outcome Y
- data.frame of covariates X
- data.frame of covariates newX

```
fit.mars <- polymars(Y, X)
out <- predict.polymars(fit.mars,
  x = as.matrix(newX))
```

Now we know how to fit the model and return predicted values, next we check out `write.SL.template` for integrating the code above into the correct syntax for `SuperLearner`

Creating wrappers

write.SL.template()

```
SL.template <- function(Y, X, newX, family,
    obsWeights, id, ...) {
  # require('pkg')
  if(family$family == 'gaussian') {  }
  if(family$family == 'binomial') {  }
  # pred is the predicted responses for newX
  pred <- numeric()
  # fit returns all objects needed for predict.SL.*
  fit <- list(object = )
  # declare class of fit for predict.SL.template
  class(fit) <- 'SL.template'
  # return a list with pred and fit
  out <- list(pred = pred, fit = fit)
  return(out)
}
}
```

Creating wrappers

Table: The arguments passed to a prediction algorithm in `SuperLearner`

Argument	Description
`Y`	the outcome variable
`X`	the training data set (the observations used to fit the model)
`newX`	the validation data set (the observations to return predictions for)
`family`	a description of the error distribution
`id`	a cluster identification
`obsWeights`	observation weights

You do not need to use all these arguments, but if you use any of them, the name must match exactly.

SL.polymars

```
My.SL.polymars <- function(Y, X, newX,
  family, ...) {
 if(family$family=="gaussian") {
  fit.mars <- polymars(Y, X)
  out <- predict.polymars(fit.mars,
   x = as.matrix(newX))
  }
  if(family$family=="binomial") {
    # insert estimation function
  }
  ... # next slide
}
```

What about `family = binomial()`?

Can leave this blank (or add `stop('only gaussian')`) if only for a specific example with continuous outcome.

To be complete, we could look up the code for a binary outcome and add this case:

```
fit.mars <- polyclass(Y, X, cv = 5)
out <- ppolyclass(cov = newX,
  fit = fit.mars)[, 2]
```

SL.polymars

```
My.SL.polymars <- function(Y, X, newX,
 family, ...) {
  if(family$family=="gaussian") {
   fit.mars <- polymars(Y, X)
   out <- predict.polymars(fit.mars,
    x = as.matrix(newX))
  }
  if(family$family=="binomial") {
   fit.mars <- polyclass(Y, X, cv = 5)
   out <- ppolyclass(cov = newX,
     fit = fit.mars)[, 2]
  }
  ... # next slide
}
```

Creating wrappers

Wrappers need to return 2 values:

1. pred: predicted `Y` values for rows in `newX`
2. fit: a list with everything needed to use `predict` method

In the `polymars` example: For the gaussian case, `predict()` needs: `object = fit.mars`
For the binomial case, `predict()` needs: `fit = fit.mars`

Note: `SuperLearner` does not use the `fit` list. If you do not plan to use the function `predict.SuperLearner` you can leave the fit object as: `fit <- vector("list", length = 0)`

SL.polymars

```
My.SL.polymars <- function(Y, X, newX,
 family, ...) {
  if(family$family=="gaussian") {
   fit.mars <- polymars(Y, X)
   out <- predict.polymars(fit.mars,
    x = as.matrix(newX))
    fit <- list(object = fit.mars)
  }
  if(family$family=="binomial") {
   fit.mars <- polyclass(Y, X, cv = 5)
   out <- ppolyclass(cov = newX,
     fit = fit.mars)[, 2]
   fit <- list(fit = fit.mars)
  }
  ... # next slide
}
```

Final step is putting everything together into a list object. The list must have 2 elements and the names must be `pred` and `fit`

Can also assign a class to the `fit` list. This will be used to look up the correct `predict` method. I'm using S3 methods here. This is only important if using `predict.SuperLearner` afterwards.

See Chambers (2008) *Software for Data Analysis* for details on S3 and S4 methods.

SL.polymars

```
My.SL.polymars  <- function(Y, X, newX,
 family, ...) {
  ... # previous slides
  out <- list(pred = pred, fit = fit)
  class(out$fit) <- c("SL.polymars")
  return(out)
}
```

Note: `out` is just a temporary variable name here.

The function should match `SL.polymars` in the **SuperLearner** package.

Creating wrappers

Important notes for creating wrappers

- Input must following naming syntax: `Y, X, ...`
- Name of new function must be different than one already in the package
- Must return a list with 2 elements named `pred` and `fit`
- `pred` must be a vector with the predicted Y values for the rows in `newX`
- `fit` can be anything if not using predict method, otherwise is a list with elements needed for `predict`

predict.SL.template

```
predict.SL.template <- function (object,
  newdata, family, X = NULL, Y = NULL, ...)
{
    pred <- numeric()
    return(pred)
}
```

predict.SL.polymars

```
predict.SL.polymars <-
function (object, newdata, family, ...) {
  if (family$family == "gaussian") {
    pred <- predict.polymars(object = object$object,
      x = as.matrix(newdata))
  }
  if (family$family == "binomial") {
    pred <- ppolyclass(cov = newdata,
      fit = object$fit)[, 2]
  }
  return(pred)
}
```

Example: creating screening algorithm

screening template

```
screen.template <- function (Y, X, family,
 obsWeights, id, ...) {
  # require('pkg')
  if (family$family == "gaussian") {
  }
  if (family$family == "binomial") {
  }
  # whichVariable is a logical vector,
  # TRUE indicates variable will be used
  whichVariable <- rep(TRUE, ncol(X))
  return(whichVariable)
}
```

Creating wrappers

Table: The arguments passed to a screening algorithm in `SuperLearner`

Argument	Description
`Y`	the outcome variable
`X`	the training data set<br>(the observations used to fit the model)
`family`	a description of the error distribution
`id`	a cluster identification
`obsWeights`	observation weights

You do not need to use all these arguments, but if you use any of them, the name must match exactly.

screen.randomForest

```
screen.randomForest <- function (Y, X,
 family, nVar = 10, ntree = 1000, ...) {
  if (family$family == "gaussian") {
    rank.rf.fit <- randomForest(Y ~ .,
     data = X, ntree = ntree)
  }
  if (family$family == "binomial") {
    rank.rf.fit <- randomForest(
     y = as.factor(Y), x = X,
     ntree = ntree)
  }
  whichVariable <- as.logical(
   rank(-rank.rf.fit$importance) <= nVar)
  return(whichVariable)
}
```

Boston Housing example

The outcome variable is the median home value (`cmedv`) for the 506 census tracts of Boston from the 1970 census.

The covariates are a mix of geographical and socioeconomic variables, like per capita crime rate (`crim`), average number of rooms per house (`rm`), distance to Boston employment centres (`dis`), indictor of tract being on the Charles river (`chas`), etc.

Boston Housing

The Boston Housing data can be found in the **mlbench** package.

Load the data

```
library(mlbench)
data(BostonHousing2)
# convert factors to numeric
BostonHousing2$chas
    <- as.numeric(BostonHousing2$chas=="1")
# select subset of variables
DATA <- BostonHousing2[, c("cmedv", "crim", "zn",
    "indus", "chas", "nox", "rm", "age", "dis",
    "rad", "tax", "ptratio", "b", "lstat")]
```

Boston Housing example

First need to decide which prediction algorithms to include in the library

Boston Housing

Algorithm	Description	Package
`glm`	linear model	**stats**
`randomForest`	random Forest	**randomForest**
`bagging`	bootstrap aggregation of trees	**ipred**
`gam`	generalized additive models	**gam**
`gbm`	gradient boosting	**gbm**
`nnet`	neural network	**nnet**
`polymars`	polynomial spline regr.	**polspline**
`bart`	Bayesian additive regr. trees	**BayesTree**
`glmnet`	elastic net	**glmnet**
`svm`	support vector machine	**e1071**
`bayesglm`	Bayesian glm	**arm**
`step`	stepwise glm	**stats**

Boston Housing

One algorithm to consider is the generalized additive model algorithm. This algorithm has a tuning parameter for the degrees of freedom in the smoother. I have set this to be 2 in `SL.gam` but we might want to consider larger values.

We could create an entirely new wrapper for `gam` and df = 3, or we can write a wrapper for the wrapper and only change the degrees of freedom value.

look at `SL.gam` to see how the degrees of freedom parameter is specified:

SL.gam

```
SL.gam <- function(Y, X, newX,
     family, obsWeights, deg.gam = 2, ...)
{
 ...   # model: Y ~ s(X, deg.gam)
       # see full functions for details
 fit.gam <- gam::gam(gam.model, data = X,
  family = family,
  control = gam.control(maxit = 50, bf.maxit = 50),
  weights = obsWeights)
 pred <- predict(fit.gam, newdata = newX,
  type = "response")
 ... # returns list here
}
```

The `SL.gam` function contains the argument `deg.gam = 2`.

Wrappers can have additional arguments, but they must have default values.

Boston Housing example

For the new wrapper, only need to change the value of `deg.gam`. Use ... to pass everything else between `SL.gam.3` and `SL.gam`.

Adjusting deg.gam in `SL.gam`

```
SL.gam.3 <- function(..., deg.gam = 3) {
  SL.gam(..., deg.gam = deg.gam)
}
```

Easy to create new wrappers by changing tuning parameter values. Check the code for the wrappers by typing the name of the function without parentheses to see what tuning parameter values are in the arguments.

Boston Housing Example

Similar to the `SL.gam` example above, the function `create.SL.glmnet` in the **SuperLearnerExtra** package can be used to create new `SL.glmnet` wrappers:

Additional wrappers

```
create.SL.glmnet(alpha = c(0.25, 0.50, 0.75))
# and set gbm to no interactions:
SL.gbm.1 <- function(...) {
  SL.gbm(..., interaction.depth = 1)
}
```

available at: `https://github.com/ecpolley/SuperLearnerExtra`

Boston Housing example

SL.library

```
SL.library <- c("SL.gam",
  "SL.gam.3", "SL.gam.4",
  "SL.gam.5", "SL.gbm.1",
  "SL.gbm", "SL.glm",
  "SL.glmnet", "SL.glmnet.0.25",
  "SL.glmnet.alpha.0.5", "SL.glmnet.0.75",
  "SL.polymars", "SL.randomForest",
  "SL.ridge", "SL.svm",
  "SL.bayesglm", "SL.step",
  "SL.step.interaction",
  "SL.bart")
```

Boston Housing example

fitSL

```
fitSL <- SuperLearner(Y = log(DATA$cmedv),
  X = subset(DATA, select = -c(cmedv)),
  SL.library = SL.library,
  family = gaussian()
)
```

```
                                Risk      Coef
SL.gam_All                0.03834031 0.0000000
SL.gam.3_All              0.03666449 0.0000000
SL.gam.4_All              0.03589859 0.0000000
SL.gam.5_All              0.03529692 0.0000000
SL.gbm.1_All              0.03040543 0.0000000
SL.gbm.2_All              0.02501729 0.0000000
SL.glm_All                0.03754472 0.0000000
SL.glmnet_All             0.03765112 0.0000000
SL.glmnet.alpha25_All     0.03754278 0.0000000
SL.glmnet.alpha50_All     0.03758802 0.0000000
SL.glmnet.alpha75_All     0.03763085 0.0000000
SL.polymars_All           0.04587432 0.0000000
SL.randomForest_All       0.02105987 0.2956277
SL.ridge_All              0.03753661 0.0000000
SL.svm_All                0.02678290 0.0000000
SL.bayesglm_All           0.03754318 0.0000000
SL.step_All               0.03753337 0.0000000
```

Boston Housing

Table: Elements of the output from `SuperLearner`

Name	Description
`SL.predict`	super learner predicted values for `newX`
`coef`	coefficient for each algorithm
`libraryNames`	names of algorithms in library
`library.predict`	matrix of predicted values for `newX` from each algorithm in the library
`cvRisk`	V-fold cross-validated risk for each algorithm in the library

The final super learner prediction model is the weighted combination of the library algorithms where the estimates of the weights can be found with `coef(fitSL)`.

To attain predictions on new observations (not in `newX`), the `predict` function will usually work. If you created new wrappers, you also need to create `predict` S3 methods for those new wrappers.

`SuperLearner` is a model selection algorithm. It does not contain a good estimate for model assessment (you could use the re-substitution method to estimate the risk but this is optimistic).

Our suggestion to assess the performance of the super learner is to run `CV.SuperLearner` (example in the next case study).

ALL EXAMPLE

- The outcome variable is an indicator of the molecular biology of the cancer tissue, either Negative or BCR/ABL.
- The sample consists of 79 individuals (42 Neg, 37 BCR/ABL).
- The data contain 2200 features (X) to be used after the filtering steps.
- Need to select algorithms appropriate for a binary outcome and a large number of covariates.

ALL

load ALL data

```
# source("http://bioconductor.org/biocLite.R")
# biocLite()
# biocLite("ALL")
library(ALL)
library(genefilter)
data(ALL)
```

The next 2 slides are the processing steps following in Gentleman, Huber and Carey (2008) “Supervised Machine Learning” in *Bioconductor Case Studies*.

```
# restrict to only the NEG and BCR/ABL outcomes
bcell <- grep("^B", as.character(ALL$BT))
moltyp <- which(as.character(ALL$mol.biol)
  %in% c("NEG", "BCR/ABL"))
ALL_bcrneg <- ALL[, intersect(bcell, moltyp)]
#drops unused levels
ALL_bcrneg$mol.biol <- factor(ALL_bcrneg$mol.biol)

# filter features
ALLfilt_bcrneg <- nsFilter(ALL_bcrneg,
  var.cutoff = 0.75)$eset
```

```r
# standardize the features
rowIQRs <- function(eSet) {
  numSamp <- ncol(eSet)
  lowQ <- rowQ(eSet, floor(0.25 * numSamp))
  upQ <- rowQ(eSet, ceiling(0.75 * numSamp))
  upQ - lowQ
}
standardize <- function(x) {
  (x - rowMedians(x)) / rowIQRs(x)
}
exprs(ALLfilt_bcrneg) <- standardize(
  exprs(ALLfilt_bcrneg))

# convert to numeric matrix for the SuperLearner
Y <- as.numeric(
  ALLfilt_bcrneg$mol.biol == "BCR/ABL")
X <- t(exprs(ALLfilt_bcrneg))
```

ALL

Possible prediction algorithms include:

- k-nearest neighbors
- elastic net (penalized regression)
- random forest

These algorithms have tuning parameters:

- `knn`: k
- `glmnet`: α
- `randomForest`: `mtry` and `nodesize`

ALL

randomForest

```
tuneGrid <- expand.grid(mtry = c(500, 1000, 2200),
  nodesize = c(1, 5, 10))

for(mm in seq(nrow(tuneGrid))) {
   eval(parse(file = "", text =
    paste("SL.randomForest.", mm,
    "<- function(..., mtry = ", tuneGrid[mm, 1],
    ", nodesize = ", tuneGrid[mm, 2], ") {
    SL.randomForest(..., mtry = mtry,
    nodesize = nodesize) }", sep = "")))
}
```

The code above is hard to follow, but I'm doing the same thing we did with `SL.gam.3` just in a `for` loop.

```
> SL.randomForest.1
function(..., mtry = 500, nodesize = 1) {
  SL.randomForest(..., mtry = mtry,
  nodesize = nodesize) }

> SL.randomForest.2
function(..., mtry = 1000, nodesize = 1) {
  SL.randomForest(..., mtry = mtry,
  nodesize = nodesize) }

> SL.randomForest.9
function(..., mtry = 2200, nodesize = 10) {
  SL.randomForest(..., mtry = mtry,
  nodesize = nodesize) }
```

ALL

Add additional `knn` wrappers using functions in **SuperLearnerExtra**

create.SL.knn

```
create.SL.knn(k = c(k = 20, 30, 40, 50))
```

---

available at: `https://github.com/ecpolley/SuperLearnerExtra`

ALL

SL.library

```
SL.library <- c("SL.knn",
   "SL.knn.20",
   "SL.knn.30",
   "SL.knn.40",
   "SL.knn.50",
   "SL.randomForest",
   "SL.glmnet",
   "SL.glmnet.0.25",
   "SL.glmnet.0.5",
   "SL.glmnet.0.75",
   "SL.mean",
   paste("SL.randomForest.",
    seq(nrow(tuneGrid)), sep = ""))
```

ALL

ALL example

```
fitSL <- SuperLearner(Y = Y, X = X,
 SL.library = SL.library, family = binomial(),
 method = "NNLS",
 cvControl = list(stratifyCV = TRUE))

fitSL
```

```
                                Risk       Coef
SL.knn_All                0.20658228 0.00000000
SL.knn20_All              0.22423347 0.00000000
SL.knn30_All              0.22299664 0.00000000
SL.knn40_All              0.23445986 0.00000000
SL.knn50_All              0.23920321 0.00000000
SL.randomForest_All       0.12418009 0.00000000
SL.glmnet_All             0.08430633 0.98039534
SL.glmnet.alpha25_All     0.10487930 0.00000000
SL.glmnet.alpha50_All     0.09331539 0.00000000
SL.glmnet.alpha75_All     0.08681511 0.00000000
SL.randomForest.1_All     0.13103528 0.00000000
SL.randomForest.2_All     0.12269094 0.00000000
SL.randomForest.3_All     0.11918439 0.00000000
SL.randomForest.4_All     0.13024104 0.00000000
SL.randomForest.5_All     0.12351049 0.00000000
SL.randomForest.6_All     0.11752733 0.01960466
SL.randomForest.7_All     0.12871385 0.00000000
```

CV.SuperLearner

```
fitSL.CV <- CV.SuperLearner(Y = Y, X = X,
  SL.library = SL.library,
  V = 20, family = binomial(),
  method = "method.NNLS",
  cvControl = list(stratifyCV = TRUE))

summary(fitSL.CV)

# can also print the LaTeX table
# requires Hmisc package
# latex(summary(fitSL.CV))
```

Algorithm	subset	Risk	SE	Min	Max
SuperLearner	–	0.101	0.020	0.003	0.382
Discrete SL	–	0.095	0.021	0.004	0.347
SL.knn(10)	All	0.212	0.018	0.070	0.460
SL.knn(20)	All	0.220	0.011	0.152	0.322
SL.knn(30)	All	0.223	0.008	0.192	0.274
SL.knn(40)	All	0.232	0.006	0.187	0.268
SL.knn(50)	All	0.238	0.004	0.218	0.260
SL.randomForest	All	0.120	0.014	0.026	0.256
SL.glmnet($\alpha = 1.0$)	All	0.088	0.022	0.002	0.395
SL.glmnet($\alpha = 0.25$)	All	0.113	0.022	0.007	0.451
SL.glmnet($\alpha = 0.50$)	All	0.106	0.023	0.004	0.447
SL.glmnet($\alpha = 0.75$)	All	0.093	0.021	0.004	0.347
SL.mean	All	0.249	0.004	0.242	0.251
SL.randomForest.1	All	0.125	0.014	0.034	0.269
SL.randomForest.2	All	0.114	0.014	0.023	0.250
SL.randomForest.3	All	0.111	0.015	0.016	0.238
SL.randomForest.4	All	0.123	0.014	0.036	0.264
SL.randomForest.5	All	0.117	0.014	0.023	0.262
SL.randomForest.6	All	0.110	0.015	0.015	0.252
SL.randomForest.7	All	0.126	0.014	0.034	0.266
SL.randomForest.8	All	0.117	0.014	0.023	0.259
SL.randomForest.9	All	0.110	0.015	0.013	0.245

van't Veer example

- 97 breast cancer patients followed for 5 years.
- Outcome is binary yes/no recur in 5 years (we do not have the date of recurrence)
- 7 clinical variables are available (age, tumor grade, etc.)
- 4348 gene expression values post-filtering

van't Veer data

The original data is available at:
`http://www.rii.com/publications/2002/vantveer.html`

One interesting "screening" is to consider the prediction algorithms on only the clinical variables or on only the gene expression variables.

screening

```
screen.clinical <- function(...){
  return(c(rep(TRUE, 7), rep(FALSE, 4348)))
}

screen.array <- function(...){
  return(c(rep(FALSE, 7), rep(TRUE, 4348)))
}
```

```
SL.library <- list(
  c("SL.knn", "All", "screen.clinical",
   "screen.corP", "screen.corP.01", "screen.glmnet"),
  c("SL.knn.20", "All", "screen.clinical",
   "screen.corP", "screen.corP.01", "screen.glmnet"),
  c("SL.glmnet", "screen.corRank.50",
   "screen.corRank.20"),
  c("SL.glmnet.0.75", "screen.corRank.50",
   "screen.corRank.20"),
  c("SL.glmnet.0.5", "screen.corRank.50",
   "screen.corRank.20"),
  c("SL.glmnet.0.25", "screen.corRank.50",
   "screen.corRank.20"),
  c("SL.randomForest", "screen.clinical",
   "screen.corP.01", "screen.glmnet"),
  c("SL.bagging", "screen.clinical",
   "screen.corP.01", "screen.glmnet"),
  c("SL.bart", "screen.clinical",
```

SuperLearner

```
fitSL <- SuperLearner(Y = surv.resp, X = X,
  SL.library = SL.library,
  family = binomial(),
  method = "method.NNLS",
  control = list(saveFitLibrary = FALSE))

fitSL
```

van't Veer

fitSL

```
                                 Risk       Coef
...
SL.knn_screen.corP.01         0.2129897 0.27517459
...
SL.glmnet_screen.corRank.20   0.2210815 0.22256164
...
SL.randomForest_clinical      0.2151708 0.08636558
...
SL.bart_clinical              0.2084039 0.41589818
```

Only presenting results for non-zero coefficients. Table does not fit on a slide.

CV.SuperLearner

```
fitSL.CV <- CV.SuperLearner(Y=surv.resp, X=X,
  V = 20,
  SL.library = SL.library,
  family = binomial(),
  method = "method.NNLS",
  cvControl = list(stratifyCV = TRUE))

summary(fitSL.CV)
```

Algorithm	subset	Risk	SE	Min	Max
SuperLearner	–	0.194	0.017	0.103	0.309
Discrete SL	–	0.238	0.024	0.127	0.415
SL.knn(10)	All	0.249	0.020	0.144	0.532
SL.knn(10)	clinical	0.239	0.019	0.138	0.496
SL.knn(10)	cor ($p < 0.1$)	0.262	0.023	0.095	0.443
SL.knn(10)	cor ($p < 0.01$)	0.224	0.020	0.088	0.365
SL.knn(10)	glmnet	0.219	0.028	0.007	0.465
SL.knn(20)	All	0.242	0.013	0.171	0.397
SL.knn(20)	clinical	0.236	0.012	0.154	0.382
SL.knn(20)	cor ($p < 0.1$)	0.233	0.017	0.108	0.342
SL.knn(20)	cor ($p < 0.01$)	0.206	0.018	0.121	0.321
SL.knn(20)	glmnet	0.217	0.026	0.018	0.405
SL.knn(30)	All	0.239	0.013	0.171	0.396
SL.knn(30)	clinical	0.236	0.012	0.169	0.386
SL.knn(30)	cor ($p < 0.1$)	0.232	0.014	0.143	0.319
SL.knn(30)	cor ($p < 0.01$)	0.215	0.017	0.136	0.346
SL.knn(30)	glmnet	0.210	0.023	0.039	0.402
SL.knn(40)	All	0.240	0.011	0.182	0.331
SL.knn(40)	clinical	0.238	0.010	0.179	0.319
SL.knn(40)	cor ($p < 0.1$)	0.236	0.012	0.166	0.316
SL.knn(40)	cor ($p < 0.01$)	0.219	0.015	0.154	0.309
SL.knn(40)	glmnet	0.211	0.021	0.060	0.346
SL.glmnet($\alpha = 1.0$)	corRank.50	0.229	0.029	0.078	0.445
SL.glmnet($\alpha = 1.0$)	corRank.20	0.208	0.026	0.048	0.424
SL.glmnet($\alpha = 0.75$)	corRank.50	0.221	0.027	0.077	0.420
SL.glmnet($\alpha = 0.75$)	corRank.20	0.209	0.026	0.046	0.421
SL.glmnet($\alpha = 0.50$)	corRank.50	0.226	0.027	0.077	0.426
SL.glmnet($\alpha = 0.50$)	corRank.20	0.211	0.026	0.059	0.419
SL.glmnet($\alpha = 0.25$)	corRank.50	0.229	0.027	0.084	0.424
SL.glmnet($\alpha = 0.25$)	corRank.20	0.216	0.025	0.072	0.406
SL.randomForest	clinical	0.198	0.019	0.098	0.391
SL.randomForest	cor ($p < 0.01$)	0.204	0.018	0.101	0.341
SL.randomForest	glmnet	0.220	0.025	0.072	0.378
SL.bagging	clinical	0.207	0.016	0.108	0.408
SL.bagging	cor ($p < 0.01$)	0.205	0.018	0.107	0.353
SL.bagging	glmnet	0.206	0.022	0.077	0.388
SL.bart	clinical	0.202	0.018	0.109	0.365
SL.bart	cor ($p < 0.01$)	0.210	0.021	0.092	0.376
SL.bart	glmnet	0.220	0.028	0.043	0.423
SL.mean	All	0.250	0.003	0.246	0.251

Installing suggested packages

```
install.packages(c("glmnet","randomForest",
  "class", "gam", "gbm", "nnet", "polspline",
  "MASS", "e1071", "stepPlr", "arm", "party",
  "spls", "LogicReg", "nnls", "multicore",
  "SIS", "BayesTree", "quadprog", "ipred",
  "mlbench", "rpart", "caret", "mda", "earth"),
  type="source",
  repos="http://cran.cnr.Berkeley.edu",
  dependencies=c("Depends", "Imports"))
# missing DSA, not available on CRAN
```

Can remove type = 'source' if system not setup to install packages from source.

Colophon

- Slides created with LaTeX package **Beamer**
- Code blocks adapted from the **tikzDevice** R package
- LaTeX package **tikz** and **sweave** for code styles
- R version 2.13.0 and **SuperLearner** version 2.0-1
- Other packages: arm 1.4-10, BayesTree 0.3-1, caret 4.88, class 7.3-3, DSA 3.1.4, e1071 1.5-25, earth 2.6-2, gam 1.04, gbm 1.6-3, glmnet 1.6, Hmisc 3.8-3, ipred 0.8-11, leaps 2.9, lme4 0.999375-39, LogicReg 1.4.10, MASS 7.3-13, mda 0.4-2, mlbench 2.1-0, modelUtils 3.1.4, nnet 7.3-1, nnls 1.3, party 0.9-9994, polspline 1.1.5, quadprog 1.5-4, randomForest 4.6-2, rpart 3.1-50, SIS 0.6,
